# Immune Recovery Following Autologous Hematopoietic Stem Cell Transplantation in HIV-Related Lymphoma Patients on the BMT CTN 0803/AMC 071 Trial

**DOI:** 10.3389/fimmu.2021.700045

**Published:** 2021-09-03

**Authors:** Polina Shindiapina, Maciej Pietrzak, Michal Seweryn, Eric McLaughlin, Xiaoli Zhang, Mat Makowski, Elshafa Hassan Ahmed, Sarah Schlotter, Rebecca Pearson, Rhonda Kitzler, Anna Mozhenkova, Jennifer Le-Rademacher, Richard F. Little, Gorgun Akpek, Ernesto Ayala, Steven M. Devine, Lawrence D. Kaplan, Ariela Noy, Uday R. Popat, Jack W. Hsu, Lawrence E. Morris, Adam M. Mendizabal, Amrita Krishnan, William Wachsman, Nita Williams, Nidhi Sharma, Craig C. Hofmeister, Stephen J. Forman, Willis H. Navarro, Joseph C. Alvarnas, Richard F. Ambinder, Gerard Lozanski, Robert A. Baiocchi

**Affiliations:** ^1^Division of Hematology, Department of Internal Medicine, The Ohio State University, Columbus, OH, United States; ^2^Comprehensive Cancer Center, The Ohio State University, Columbus, OH, United States; ^3^Department of Biomedical Informatics, The Ohio State University, Columbus, OH, United States; ^4^Biobank Lab, Department of Molecular Biophysics, Faculty of Biology and Environmental Protection, University of Łódź, Łódź, Poland; ^5^Emmes Company, Rockville, MD, United States; ^6^Department of Veterenary Biosciences, College of Veterenary Medicine, The Ohio State University, Columbus, OH, United States; ^7^College of Medicine, The Ohio State University, Columbus, OH, United States; ^8^Department of Pathology, The Ohio State University, Columbus, OH, United States; ^9^Division of Clinical Trials and Biostatistics, Department of Quantitative Health Sciences, Mayo Clinic, Rochester, MN, United States; ^10^National Cancer Institute, National Institutes of Health, Bethesda, MD, United States; ^11^Pacific Central Coast Health Centers, San Luis Obispo, CA, United States; ^12^Department of Internal Medicine, Hematology & Oncology, Mayo Clinic, Jacksonville, FL, United States; ^13^Center for International Blood and Marrow Transplant Research, National Marrow Donor Program/Be The Match, Minneapolis, MN, United States; ^14^Department of Medicine, University of California, San Francisco, San Francisco, CA, United States; ^15^Department of Medicine, Memorial Sloan Kettering Cancer Center and Weill Cornell Medical College, New York, NY, United States; ^16^Department of Stem Cell Transplantation and Cellular Therapy, University of Texas, MD Anderson Cancer Center, Houston, TX, United States; ^17^Division of Hematology and Oncology, Department of Medicine, University of Florida, Gainesville, FL, United States; ^18^Blood and Marrow Transplant Program at Northside Hospital, Atlanta, GA, United States; ^19^Department of Hematology & Hematopoietic Cell Transplantation, City of Hope Comprehensive Cancer Center, Duarte, CA, United States; ^20^Moores University of California San Diego Cancer Center, La Jolla, CA, United States; ^21^Veterans Affairs San Diego Healthcare System, San Diego, CA, United States; ^22^Winship Cancer Institute, Emory University, Atlanta, GA, United States; ^23^Division of Hematology/Oncology/Transplantation, University of Minnesota, Minneapolis, MN, United States; ^24^Global Research and Development, Atara Biotherapeutics, Inc., San Francisco, CA, United States; ^25^Division of Hematologic Malignancies, Sidney Kimmel Comprehensive Cancer Center (SKCCC), Johns Hopkins Medical Institutions, Baltimore, MD, United States

**Keywords:** human immunodeficiency virus (HIV), hematopoeietic stem cell transplantation, Hodgkin lymphoma (HL), Non-Hodgkin lymphoma, multiple myeloma

## Abstract

We report a first in-depth comparison of immune reconstitution in patients with HIV-related lymphoma following autologous hematopoietic cell transplant (AHCT) recipients (n=37, lymphoma, BEAM conditioning), HIV(-) AHCT recipients (n=30, myeloma, melphalan conditioning) at 56, 180, and 365 days post-AHCT, and 71 healthy control subjects. Principal component analysis showed that immune cell composition in HIV(+) and HIV(-) AHCT recipients clustered away from healthy controls and from each other at each time point, but approached healthy controls over time. Unsupervised feature importance score analysis identified activated T cells, cytotoxic memory and effector T cells [higher in HIV(+)], and naïve and memory T helper cells [lower HIV(+)] as a having a significant impact on differences between HIV(+) AHCT recipient and healthy control lymphocyte composition (p<0.0033). HIV(+) AHCT recipients also demonstrated lower median absolute numbers of activated B cells and lower NK cell sub-populations, compared to healthy controls (p<0.0033) and HIV(-) AHCT recipients (p<0.006). HIV(+) patient T cells showed robust IFNγ production in response to HIV and EBV recall antigens. Overall, HIV(+) AHCT recipients, but not HIV(-) AHCT recipients, exhibited reconstitution of pro-inflammatory immune profiling that was consistent with that seen in patients with chronic HIV infection treated with antiretroviral regimens. Our results further support the use of AHCT in HIV(+) individuals with relapsed/refractory lymphoma.

## Introduction

Improved outcomes in treatment of HIV-associated lymphomas have been achieved in the era of combined antiretroviral therapy [cART] by using multi-agent, dose-intense immuno-chemotherapeutic regimens, suggesting that HIV-positive [HIV(+)] patients can tolerate intensive approaches with curative intent (1–6). Standard combination therapeutic approaches have achieved complete response (CR) rates >70% in HIV-associated Hodgkin’s (HL) and non-Hodgkin’s lymphoma (NHL) patients (7–10). However, not all HIV(+) patients with HL and NHL achieve a sustained first complete remission. In HIV-related NHL, the risk of relapse exceeded 11% after a median follow up of 4.5 years in patients that achieved CR with initial chemotherapy in a prospective observational study of 254 patients (11).

Multiple groups have performed autologous hematopoieticcell transplant (AHCT) in limited numbers of HIV(+) patients with high-risk lymphoma in the era of HIV viral load control with cART. Observational studies have reported successful engraftment and low risk of post-transplant infection (12–16). BMT CTN 0803/AMC 071/NCT01141712 was the first interventional, Phase 2 prospective trial investigating the safety and efficacy of a unified myeloablative conditioning regimen followed by AHCT in 40 patients with controlled HIV infection and chemotherapy sensitive relapsed/refractory HIV-associated lymphoma. This study demonstrated two-year progression-free survival (PFS) of 79.8% and a low 1-year transplant-related mortality (TRM) of 5.2% (17). Finally, a prospective multicenter study of AHCT for early consolidation in 16 patients with high or high–intermediate risk HIV-related NHL showed a 50-month OS and PFS of 93.7% (18).

Few studies to date have documented the kinetics of immune system recovery post-transplant in HIV(+) patients, reporting delayed but successful recovery of CD4+ T cells and elevated CD8+ T cell counts by 1-2 years post-transplant (13, 16–18). Furthermore, few comparisons of immune reconstitution after autologous transplant following different conditioning regimens exist. We hypothesized that immune reconstitution post-ASCT is influenced by multiple factors, including the type of hematologic malignancy, the choice of chemotherapy conditioning regimen and the presence of immune stimuli, such as a chronic HIV infection or vaccination. Here we describe a first detailed analysis of quantitative and functional recovery of the cellular adaptive and innate immunity in HIV(+) recipients of AHCT for treatment of HL or NHL on the BMT CTN 0803/AMC 071/NCT01141712 trial. This was compared to the kinetics of immune reconstitution in a cohort HIV(-) AHCT recipients with multiple myeloma (MM) that received a pneumococcal polyvalent vaccine on an observational clinical trial (NCT00569309), and healthy controls. Comparisons were made across total numbers and multiple sub-sets of T cells, B cells and NK cells obtained by 5-color flow cytometry. Global trends in immune reconstitution were compared by principal component analysis (PCA). Lymphocyte populations that had the greatest impact on the difference in immune reconstitution between subject cohorts were identified by feature importance score analysis (FIS), a random sampling analysis aimed at the quantification of importance of specific immune cell populations to the difference between the cohorts. Finally, direct comparisons of reconstitution of specific lymphocyte subsets were performed using Wilcoxon rank-sum tests with false discovery rate (FDR) correction.

We found that immune reconstitution followed distinct avenues in HIV(+) and HIV(-) AHCT recipients, with HIV(-) cohort achieving similarity with healthy controls and HIV(+) cohort reconstituting immune features associated with chronic, controlled HIV infection at 1 year post-transplant. Within the first year following AHCT, we observed persistently lower absolute numbers of CD4+ helper T cell subsets, higher numbers of CD8+ T cell subsets and activated T cells, lower numbers of activated B cells and lower numbers of natural killer (NK) cell subsets expressing marker of activation and inhibition in HIV(+) AHCT recipients, compared to HIV(-) AHCT recipients and healthy controls. T lymphocytes from HIV(+) patients responded robustly to recall antigen challenge with HIV and Epstein-Barr virus (EBV) pepmixes. Overall, these data indicate a trend toward an activation-prone state of the adaptive cellular immunome in HIV(+) patients after AHCT that is maintained despite undetectable HIV viral load in the majority of patients, and despite response of their lymphoma to myeloablative therapy. Our findings suggest that HIV(+) individuals in post-transplant remission from aggressive lymphoma may continue to experience impaired immune surveillance associated with chronic HIV. Our conclusions warrant further investigation into this distinct immune reconstitution and long-term clinical consequences in this patient population.

## Methods

### Flow Cytometry Acquisition and Analysis

Whole blood samples were collected in anticoagulant citrate dextrose solution (BD Medical Technology) from 37 HIV(+) AHCT recipients, 30 HIV(-) AHCT recipients and 71 healthy controls. Five color flow cytometry was performed using defined antibody panels as previously described (19). Antibody vendors and clones are listed in **Table S4**. Briefly, flow cytometry was performed *via* stain lyse run approach on automated Prep2 Workstation cell preparation system and TQ-prep Workstation with ImmunoPrep Reagent System, according to the manufacturer’s instructions (Beckman Coulter). Acquisition and analysis were performed using CXP software with Prim Plot utility, using CD45 and side scatter characteristics to gate on lymphocytes. The results were expressed as absolute number or percentage of total gated lymphocytes and absolute number of cells per microliter of blood using absolute lymphocytes count from ActDiff hematology analyzer (Beckman Coulter).

### Statistics

#### Unsupervised Principal Component Analysis

Flow cytometry data on cell proportions from HIV(+) AHCT recipients, HIV(-) AHCT recipients and healthy controls were compared by principal component analysis (PCA) in R software across 18 mononuclear immune cell subsets. HIV(+) AHCT recipients and healthy controls were also compared using 100 mononuclear immune cell subsets.

#### Importance Index and Feature Importance Score Analysis

A pairwise analysis of immune profile similarity was performed using I-Index, an overlap measure defined by Rempala and Seweryn, in R software using the R-package “divo” (available from URL https://CRAN.R-project.org/package=divo) (20). The pairwise similarity matrix was used for hierarchical clustering as described (21).

The immune data are sparse and inter-correlated due to the presence of highly abundant immune populations in conjunction with low cell numbers across other immune populations. Immune cell subsets are also highly correlated across multiple samples and subject cohorts (21). Therefore, to identify the cellular subsets that had the most impact on differences in immune reconstitution observed in HIV(+) AHCT recipients and healthy controls, we applied a pre-filtering approach to increase the power of the study and avoid redundant comparisons, as previously described (20, 21). This method guarantees an extremely low likelihood of false positives and a high rate of identification of true positives (21). In this approach, we use an information-theoretic similarity measure (I-index), which is able to detect overlap between two sets of immune cell populations derived from distinct subject cohorts in the setting of highly abundant as well as rare immune cell populations (20). The core of the method is presented in the supplemental **Figure S1**.

Below, we present a short description of our approach applied to compare two sets of lymphocyte characteristics (‘case’ and ‘control’) (21). Briefly, after data pre-processing and normalization, cohort lymphocyte characteristics were compared as follows: each individual ‘case’ immune profile was compared with the set of all ‘control’ immune profiles. This defines a distance ‘d’ between each case immune profile and a set of control samples d(case; controls) = [I-index(case + controls)] – [I-index(controls)]. The main aim was to quantify the importance of specific immune cell populations to the difference between “case” and “control” immune profiles. We used a previously described approach: for a lymphocyte population ‘j’ and a defined subset of immune cell populations ‘J’, which contains ‘j’, the feature importance score (FIS) for lymphocyte population ‘j’ was defined as the contribution of ‘j’ to the distance between case and control lymphocyte sets (21). This contribution was computed as the difference based on the whole set ‘J’ versus the set ‘J-j’; ‘J’ and ‘J-j’ of “cases” were compared to “controls”. This makes the FIS for feature ‘j’ dependent on the set ‘J’. Hence, we randomly selected 500,000 subsets of 10 immune marker combinations from the 100 marker combination set and 7 immune marker combinations from the 18 marker immune marker set. For each feature in these sets, we calculate its FIS for the comparison between any ASCT recipient immune cell characteristics and the set of controls. Subsequently, we calculate the median FIS for each feature/ASCT recipient combination and used the Wilcoxon test (with FDR correction) to find immune marker combinations with positive FIS in ‘almost all’ ASCT recipients (21).

#### Statistical Analysis Comparing Distributions of Absolute Cell Numbers and Cell Proportions of Specific Immune Cell Subsets

Flow cytometry results for immune cell subset recovery in HIV(+), HIV(-) and healthy control cohorts were compared by Wilcoxon rank sum tests with Bonferroni correction. For the 18 immune cell population comparison of HIV(+) AHCT recipients, HIV(-) AHCT recipients and healthy control, false discovery rate was controlled for, so significant differences were indicated by p<0.006 (1/162 comparisons=0.006). Similarly for the 100 immune cell population panel comparison of HIV(+) AHCT recipients and healthy controls, significant differences were indicated by p<0.0033 (1/300 comparisons=0.0033). For the comparisons between HIV(+) AHCT recipients with Hodgkin’s lymphoma and HIV(-) AHCT recipients across the 18 immune cell population panel, controlling the false discovery rate gave a significance cutoff a p<0.0185 (1/54 comparisons).

#### Functional Immune Recovery Analysis

PBMCs were isolated from blood of HIV(+) AHCT recipients collected at 56, 180, and 360 days post AHCT (n=31 at days 56 and 180, n=22 at day 365), and 5 healthy volunteers by density gradient centrifugation. Secretion of interferon-gamma (IFNγ) in response to pepmixes containing epitopes of the EBV recall antigen BZLF1, HIV recall antigen GAG or CD3-specific antibody was assessed with the Human IFNγ enzyme-linked immunospot (ELISpot) kit (Mabtech) as per manufacturer’s instructions. Spots were visualized and counted using an Immunospot Imaging Analyzer (Cellular Technology Ltd., Cleveland, OH).

#### Study Approval

This is a correlative study that used de-identified samples from patients enrolled on the BMT CTN 0803/AMC 071/NCT01141712 and NCT00569309 trials. Multiple institutions recruited patients onto the BMT CTN 0803/AMC 071/NCT01141712 trial. In each institution, patient enrollment was approved by the Institutional Review Board in accordance to the ethical principles regarding human experimentation stated in the Declaration of Helsinki. Written informed consent was received at each individual institution from participants prior to inclusion in the study. De-identified blood samples were shipped to the Ohio State University for analysis that incorporated secondary use of de-identified data and did not constitute human subjects research. The following institutions recruited patients onto the BMT CTN 0803/AMC 071/NCT01141712 trial: Beth Israel Deaconess Medical Center, Blood & Marrow Transplant Program at Northside Hospital, City of Hope National Medical Center, Emory University, Fred Hutchinson Cancer Research Center, H. Lee Moffitt Cancer Center, Johns Hopkins Sidney Kimmel Comprehensive Cancer Center, Memorial Sloan-Kettering Cancer Center, Ohio State University Arthur G. James Cancer Hospital, Rush University Medical Center, University of California Los Angeles, Moores University of California San Diego Cancer Center, University of California San Francisco, University of Florida Shands Medical Center, University of Maryland Medical Systems – Greenebaum Cancer Center, University of Rochester Medical Center, University of Texas MD Andersen Cancer Research Center, Washington University Barnes-Jewish Hospital, and New York-Presbyterian Hospital/Weill Cornell Medical Center. The Ohio State University recruited patients onto the NCT00569309 trial, and use of de-identified data was approved by the Ohio State University Institutional Review Board (Study Number: 2021C0016). Normal ranges for cell subsets were established by the clinical pathology laboratory using de-identified samples from normal donors according to clinical laboratory regulations.

## Results

### Demographics of Subject Cohorts

Demographic information on HIV(+) AHCT recipients, HIV(-) AHCT recipients and healthy controls is shown in **Table 1**. The HIV(+) cohort consisted of 37 patients, 14 of whom had HL and 23 had NHL, that received ASCT on the BMT CTN 0903/AMC 071/NCT01141712 phase II study after myeloablative conditioning with carmustine, cytarabine, etoposide and melphalan (BEAM) (17). Clinical characteristics of the HIV(+) AHCT-recipient cohort have been previously described (17). The HIV(-) AHCT recipient cohort included subjects with multiple myeloma that received myeloablative conditioning with either 140 or 200 mg/m2 melphalan enrolled in an observational study (NCT00569309) investigating immune reconstitution in patients that received pneumococcal polyvalent vaccine intramuscularly once in weeks 9, 17, and 25 after AHCT. HIV(-) ASCT recipients had no exposure to other malignancy-directed treatments following AHCT, achieved an absolute neutrophil count ≥1000/μL and an absolute platelet count ≥ 75,000/μL by week 9 post-AHCT, and achieved CR in 44% of patients and overall responses (OR) in of 94% of patients 60 days post-AHCT. Blood samples from both ASCT recipient cohorts were collected at days 56, 180 and 365 from HIV(+) and HIV(-) AHCT recipients. One sample per individual volunteer was collected for the HIV-negative healthy control cohort.

**Table 1:** Demographic characteristics of the patient cohorts.

Characteristic	HIV(+) AHCT recipients, n (%)	HIV(-) AHCT recipients, n (%)	Healthy controls, n (%)
**N**	37	30	71
**HIV status**			
**HIV(+)**	37	0	0
** HIV(-)**	0	30	71
**Disease**			
** Multiple myeloma**	0	30 (100)	0
** Hodgkin’s lymphoma**	14 (38)	0	0
** Diffuse large B cell lymphoma**	14 (38)	0	0
** Burkitt’s lymphoma**	7 (19)	0	0
** Plasmablastic lymphoma**	2 (5)	0	0
**Age**			
** mean (SD)**	48 (9)	51.4 (11)	42 (11.7)
** median (min – max)**	46 (22-62)	52.5 (18-71)	49 (21-68)
**Gender**			
** Male**	32 (86%)	17 (57)	21 (30%)
** Female**	5 (14%)	13 (43)	49 (70%)
**Race/Ethnicity**			
** Black**	8 (22)	4 (13)	5 (7%)
** White**	26 (70)	26 (87)	65 (91%)
** Other**	3 (8)	0	1 (1%)
**Conditioning chemotherapy**			
** BEAM**	37 (100)	0	NA
**melphalan 140 mg/m2**	0	5 (17)	NA
** melphalan 200 mg/m2**	0	25 (83)	NA
**Cell dose (CD34, 10^6^/kg)**			
** mean (SD)**	4.7 (2.6)	5.0 (2)	NA
** median (min – max)**	4 (1.6-11.0)	4.7 (2.0-13)	NA
**Best overall response pre-transplant**			
** CR**	30 (81)	5 (17)	NA
** PR**	5 (14)	NA	NA
** RD or PD**	2 (5)	NA	NA
** VGPR**	NA	12 (40)	NA
** PR/MR/SD**	NA	12 (40)	NA
** NA**	0	1 (3.3)	NA
**Prior lymphoma therapies**			
** 1 induction regimen**	37 (100)	NA	NA
** 0 salvage regimens**	4 (11)	NA	NA
** 1 salvage regimen**	27 (73)	NA	NA
** 2 salvage regimens**	5 (14)	NA	NA
** unknown salvage**	1 (2)	NA	NA
**Prior myeloma therapies (excluding radiation)**			
** 1 MM regimen**	NA	20 (67)	NA
** 2 MM regimens**	NA	6 (20.0)	NA
** 3-5 MM regimens**	NA	4 (13)	NA

CR, complete response; PR, partial response; RD, relapsed disease; PD, progressive disease; MM, multiple myeloma; SD, standard deviation; NA, not applicable.

### Global Comparison of Immune Reconstitution in HIV(+) and HIV(-) Patients Following AHCT

**Figure 1:**
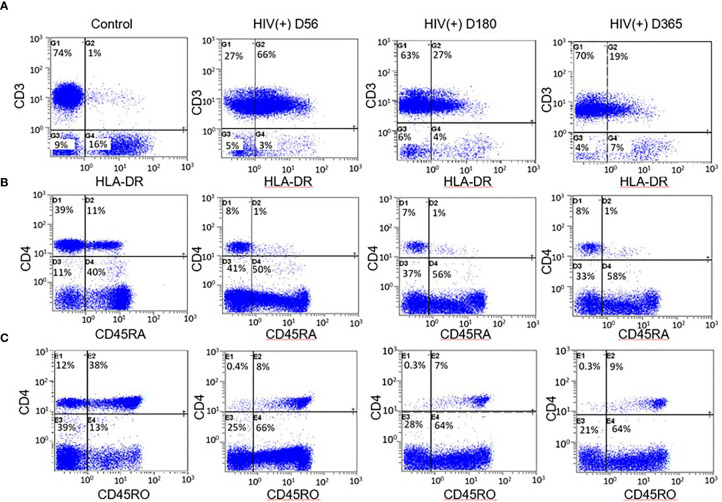
Example flow cytometry comparison between HIV(+) AHCT recipient and control. **(A)** Percentage of CD3+ lymphocytes expressing the activation marker HLA-DR in healthy control and HIV(+) AHCT recipient on days 56, 180 and 365 post-transplant. **(B)** Percentage of CD4+ lymphocytes expressing the naïve cell marker CD45RA in healthy control and HIV(+) AHCT recipient on days 56, 180 and 365 post-transplant. **(C)** Percentage of CD4+ lymphocytes expressing the memory cell marker CD45RO in healthy control and HIV(+) AHCT recipient on days 56, 180 and 365 post-transplant.

**Figure 2:**
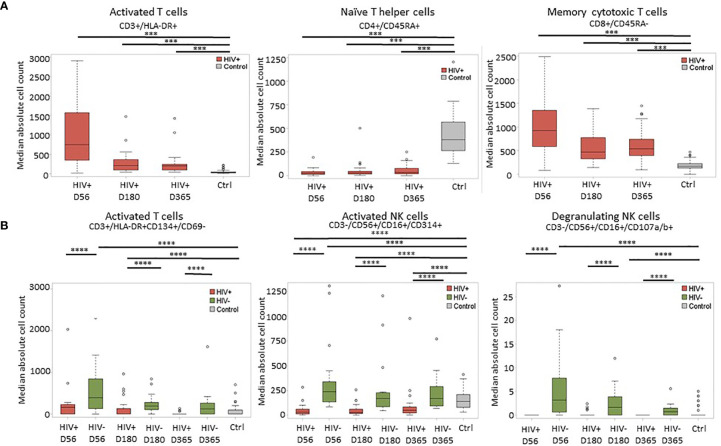
Representative plots of median absolute cell numbers determined to be significant by the Wilcoxon-rank sum test. **(A)** Comparison between HIV(+) AHCT recipients and healthy controls at days 56, 180 and 365 post-transplant; horizontal lines with *** indicate comparisons with p < 0.0007. **(B)** Comparison of HIV(+) and HIV(-) immune features to healthy controls at days 56, 180 and 365 post-transplant; horizontal lines with **** indicate comparisons with p < 0.002.

**Figure 3:**
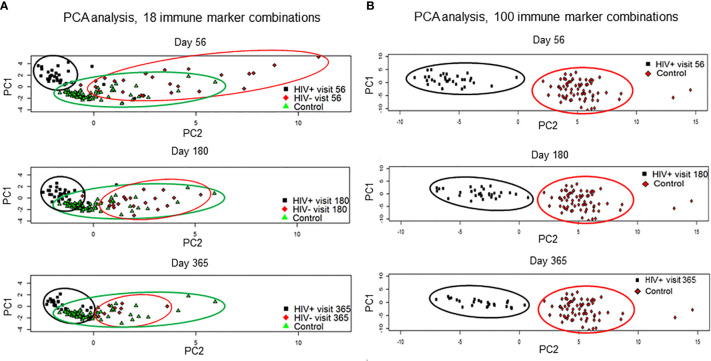
Results of the principal component analysis by visits at day 56, 180 and 365. **(A)** HIV(+) AHCT recipient, HIV(-) AHCT recipient and healthy control lymphocyte populations were compared across 18 immune marker combinations at days 56, 180 and 365 post-transplant. **(B)** HIV(+) and healthy control lymphocyte populations were compared across 100 immune marker combinations at days 56, 180 and 365 post-transplant.

Immune reconstitution was analyzed by 5 color flow cytometry. HIV(+) AHCT recipients were compared to controls across 100 immune cell populations. Representative flow cytometry data showing an evolution of reconstitution of T lymphocyte subsets from an individual HIV(+) patient, compared to a healthy control subject, is shown in **Figure 1**. At all time points, the HIV(+) AHCT recipients had a higher proportion of activated CD3+/HLA-DR+ T cells (**Figure 1A**), and lower proportions of naïve CD4+/CD45RA+ T cells (**Figure 1B**) and memory CD4+/CD45RO+ T cells (**Figure 1C**). Comparisons of median absolute cell numbers demonstrated appreciable differences between subject cohorts. HIV(+) AHCT recipients showed higher absolute median activated T cells (CD3+/HLA-DR+, p-value <0.0001 at all time points), lower naïve CD4+ T cells (CD4+/CD45RA+, p-value <0.0001 at all time points) and higher memory cytotoxic T cells (CD8+/CD45RA-, p-value <0.0001 at all time points) following AHCT, compared to healthy controls (**Figure 2A**). Additional immune populations demonstrating significant differences in HIV(+) ASCT recipients compared to healthy controls are shown in **Table S1**. Principal component analysis (PCA) comparison of HIV(+) AHCT recipients and healthy controls across 100 immune cell populations showed that cohort and control immune characteristics approached each other over time, but did not overlap by Days 180 or 365 (**Figure 3B**).

Significant differences were similarly identified in a three-way comparison of median absolute immune cell numbers in HIV(+) AHCT recipients, HIV(-) AHCT recipients and healthy controls. This comparison was performed across 18 immune cell populations. The median absolute number of late-activated T cells (CD3+/HLA-DR+CD134+/CD69-) was significantly higher in HIV(-) AHCT recipients compared to both HIV(+) AHCT recipients and healthy controls on Days 56 and 180 post-transplant (**Figure 2B** and **Table S2**). Significant differences between the HIV(-) AHCT recipient and healthy control cohorts resolved by Day 365, but the difference between HIV(+) AHCT recipients and HIV(-) AHCT recipients remained significant on Day 365, indicating that these subject cohorts diverged in reconstitution of this lymphocyte population. Median absolute numbers of activated NK cells (CD3-/CD56+/CD16+/CD314+) were significantly lower in HIV(+) AHCT recipients compared with HIV(-) AHCT recipients and healthy controls on Day 56 post-transplant, while the numbers in HIV(-) AHCT recipients significantly exceeded those in HIV(+) AHCT recipients and healthy controls (**Figure 2B**). Significant differences between the HIV(-) AHCT recipient and healthy control cohort resolved by Day 180, but the differences between HIV(-) and HIV(+) AHCT recipients, and HIV(+) AHCT recipients and healthy controls persisted throughout the post-transplant year. Similarly, NK cells expressing the degranulation marker (CD3-/CD56+/CD16+/CD107a/b+) were significantly higher in HIV(-) AHCT recipients compared to healthy controls on days 56 and 180 post-transplant, and remained significantly higher than in HIV(+) AHCT recipients throughout the post-transplant year (**Figure 2B**). Please see **Tables S1** and **S2** for individual p-values for the significantly different comparisons. These data illustrate that differences between the HIV(+) AHCT recipient, HIV(-) AHCT recipient and healthy control cohorts diminished over time but remained appreciable throughout the post-AHCT year.

PCA analysis comparing reconstitution of percent prevalence of 18 immune cell subsets showed that HIV(+) and HIV(-) AHCT recipients clustered away from healthy controls and away from each other at Day 56 (**Figure 3A**). HIV(+) and HIV(-) immune features approached each other and healthy controls by Day 365. Both HIV(+) and HIV(-) AHCT recipient immune characteristics partially overlapped with those of healthy controls, but remained distinct from each other at 1 year post-transplant.

Taken together, these results demonstrate that both HIV(+) and HIV(-) AHCT recipient immune reconstitution approached healthy controls and each other over time, but retained appreciable global differences throughout the 1 year post-AHCT period.

### Differences in Specific Immune Cell Subsets in HIV(+) AHCT Recipients and Healthy Controls

Distributions of absolute numbers of 100 specific immune cell subsets in HIV(+) AHCT recipients and healthy controls were compared by Wilcoxon rank sum test at each post-AHCT time point. **Table 2** shows broad categories of immune cell subsets under study. **Table S1** shows results summarizing the differences in specific subsets within each broad category in HIV(+) AHCT recipients and healthy controls (100 subsets).

**Table 2:** Immune marker combinations and corresponding function of cellular subsets that were identified as significantly contributing to the differences between case and control lymphocyte populations by the FIS analysis.

Immune marker combination	Function
CD3+/HLA-DR+	Activated T cells that express the late activation marker HLA-DR
CD3+/HLA-DR+/CD69+/CD134-	Activated T cells that express the early activation marker CD69 and late activation marker HLA-DR and lack activation marker CD134
CD4+/CD45RA+	Naïve T helper cells
CD4+/CD45RO+	Memory T helper cells
CD4+/CD27+	Naïve and memory T helper cells
CD8+/CD45RA-	Memory cytotoxic T cells
CD8+/CD45RO+	Memory cytotoxic T cells
CD8+/CD27-	Effector cytotoxic T cells
CD27-/CD45RO+	Central memory and effector memory lymphocytes
CD4+/CD29-	T helper cell subset that lack beta(1) integrin CD29
CD8+/CD45RA-/CD29+	Memory cytotoxic T cells
CD8+/CD45RO+/CD29+	Memory cytotoxic T cells
CD8+/CD29+	Memory cytotoxic T cells
CD4+/CD127+	Naïve, central memory and effector memory T helper cells
CD8+/CD25-	Cytotoxic T cell subset that lacks the intermediate activation marker CD25
Total CD8+	Total cytotoxic T cells
CD8+/CD127-	Effector cytotoxic T cells
CD3-/CD56+/CD16+/CD107a/b+	Cytotoxic NK cells expressing marker of degranulation
CD3-/CD56+/CD16+/CD134+	Cytotoxic NK cells expressing marker of stimulation

To summarize, multiple subsets of total T lymphocytes, T helper and cytotoxic T cells, naïve and memory T cells, NK cells and B cells showed significant differences between the two cohorts (p<0.0033). At 1 year post-AHCT, HIV(+) AHCT recipients showed higher absolute numbers of activated CD3+ T cells expressing activation markers HLA-DR with or without CD69; higher numbers of CD8+ cytotoxic T cell subsets; higher numbers central memory and effector memory lymphocytes CD27-/CD45RO+; and higher numbers of CD45RA+ /CD45RO+ double positive cells that may represent a transitional population between naïve and memory T lymphocytes (**Table S1**). HIV(+) AHCT recipients showed significantly lower absolute numbers of CD3+ T cells expressing the activation marker CD134; lower numbers of CD4+ T helper cell subsets; and lower numbers naïve T cells and the memory cell subset CD29-/CD45RO+ at 1 years post-AHCT.

HIV(+) AHCT recipients also showed lower absolute numbers of activated CD19+/CD80+ B cells and lower numbers of CD3-/CD56+ NK cell subsets (**Table S1**). NK cell subsets that were significantly lower in HIV(+) AHCT recipients compared to healthy controls at 1 year post-AHCT included cytotoxic NK cells expressing the inhibitory C-type lectin receptor NKG2A (CD3-/CD56+/CD16+/CD159a+); regulatory NK cells expressing the lysosomal-associated membrane protein 3 (CD63, also known as LAMP-3) that is a marker of exocytosis (CD3-/CD56+/CD16-/CD63+); cytotoxic NK cell subsets expressing the activating C-type lectin receptor NKG2D (CD314) with or without CD63(CD3-/CD56+/CD16+/CD314+ and CD3-/CD56+/CD16+/CD63+/CD314+); and mature cytotoxic NK cells lacking the differentiation marker CD117 (CD16+/CD56+/CD3-/CD117-). Activated regulatory NK cells (CD3-/CD56+/CD16-/CD314+) represented the only NK cell subset that was elevated in the HIV(+) AHCT recipient cohort compared to healthy controls at 1 year.

Clinical outcomes in the HIV(+) AHCT recipients that underwent transplantation on BMT CTN 0803/AMC 071 have been previously reported, with 22 of 40 (55%) AHCT recipients experiencing at least 1 infectious episode, and 9 of 40 patients (22.5%) experiencing severe grade 3-5 adverse events post-transplant (17). There were no significant differences identified during a Wilcoxon rank sum test comparison with the Kruskal-Wallis test with false discovery rate (FDR) correction of the absolute cell numbers and reconstitution kinetics between HIV(+) ASCT recipients that experienced infectious adverse events were compared to the HIV(+) subjects that remained infection-free post-transplant (not shown). Similarly, no significant differences were identified when HIV(+) with significant adverse events were compared to those without grade 3-5 adverse events (not shown).

**Figure 4:**
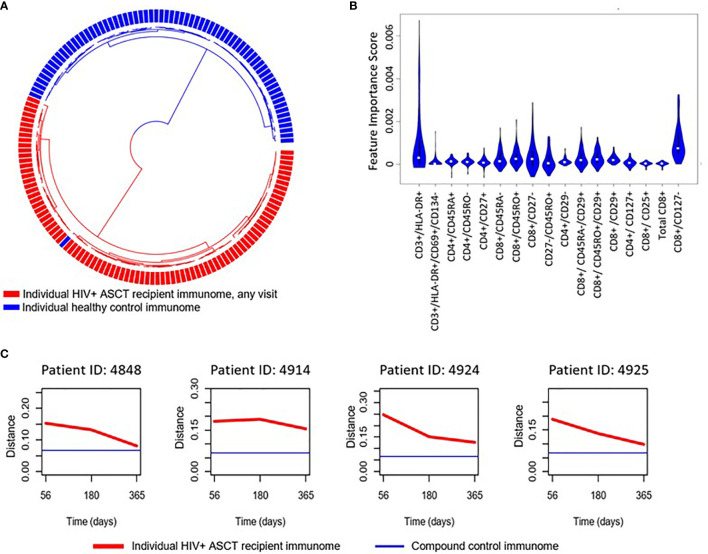
**(A)** Circularized dendrogram shows results of the I-index analysis of HIV(+) AHCT recipients (all visits) and controls: distance between individual cases is proportional to the degree of difference between patient and control lymphocyte populations. **(B)** Violin plot shows the impact of specific immune subsets on the degree of difference between HIV(+) (all visits) and control cohorts. Immune cell subsets with significant FIS are shown. **(C)** Evolution of the distance of randomly selected HIV(+) ASCT recipients’ lymphocyte populations (red line) to a set of compound control lymphocyte populations (blue line), where distance from control is defined as 1 - I-index.

We implemented an independent, unsupervised random sampling analysis using absolute immune cell numbers to identify the cellular subsets that had the most definitive impact on the difference between HIV(+) AHCT recipient and healthy control lymphocyte populations. The importance of each immune marker combination was defined as its contribution to the similarity between a given HIV(+) AHCT recipient lymphocyte features and the set of healthy control lymphocyte features in a large number of random sampling comparisons (21). To validate this approach for our subject cohorts, we used it to compare the lymphocyte composition of HIV(+) AHCT recipients to healthy controls (**Figure 4A**). A circularized dendrogram shows the results of the analysis of HIV(+) AHCT recipients (all time points) and healthy controls, where the spatial separation between individual patient and control cases is proportional to the degree of difference between HIV(+) AHCT recipients and healthy control lymphocytes. The dendrogram shows that HIV(+) AHCT recipients segregated away from 70 of 71 healthy control lymphocyte compositions, consistent with the results of the PCA analyses. To further refine these results and reduce false positives, we quantified the impact of a single lymphocyte populations in the context of various immune marker combinations on the difference between the HIV(+) AHCT recipient and healthy control lymphocytes. To this aim, we used an approach described by Pietrzak and co-authors and calculated a feature importance score (FIS) for each cell type (21). Subsequently, the FDR correction was used to identify 17 immune marker combinations with FIS values that were significantly greater than zero for the comparison between HIV(+) AHCT recipient and healthy control cohorts. A violin plot showing the magnitude of the impact of individual immune cell subsets on the difference of HIV(+) AHCT recipient lymphocytes from healthy controls is shown in **Figure 4B**. Functions of each immune cell subset identified as having significantly different FIS values are shown in **Table 2**.

Multiple T cell subsets (**Figure 4B**) were identified as significantly different between HIV(+) AHCT recipients and healthy controls by FIS values: T cells expressing markers of activation (CD3+/DLA-DR+ and CD3+/HLA-DR+/CD69+/CD134-); naïve CD4+ T helper cells (CD4+/CD45RA+ and CD4+/CD45RO-), CD4+/CD27+ T helper cells that could belong to the naïve or memory cell compartments; CD4+ T helper cells lacking the beta integrin CD29; CD4+ effector T cells (CD4+/CD27-) and CD4+ T helper cells expressing the IL-7 receptor CD127 that may belong to the naïve, central memory or effector memory compartments. Subsets of cytotoxic CD8+ T cells were also identified, including total CD8+ T cells; activated CD8+/CD25 cytotoxic T cells; memory cytotoxic T cells (CD8+/CD45RO+, CD8+/CD45RA-, CD8+/CD45RO+/CD29+ and CD8+/CD45RA-/CD29+); as well as cytotoxic effector T cells (CD8+/CD127- and CD8+/CD27-). Overall, activated T cells, cytotoxic T cells, T helper cells, naïve and memory T cell subsets appear to be the most impactful drivers of the global differences between the HIV(+) AHCT recipient and healthy control lymphocytes.

Furthermore, analysis of similarity of individual HIV(+) AHCT recipients to the compound healthy control lymphocyte features, where distance between the sets of immune characteristics was defined as [1 - I-index], showed that lymphocyte characteristics of individual HIV(+) patients approached healthy controls over time, but never overlapped (**Figures 4C** and **S2**).

**Figure 5:**
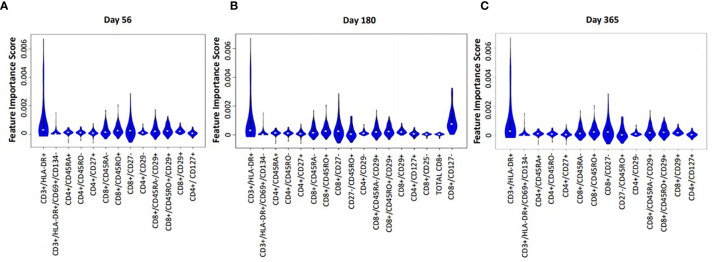
Violin plot shows the impact of specific immune subsets on the degree of difference between HIV(+) and control cohorts at days 56 **(A)**, 180 **(B)** and 365 **(C)** post-transplant.

Median absolute cell numbers of HIV(+) AHCT recipients and healthy controls were compared by FIS analysis across 100 immune marker combinations at each time point following AHCT. Immune cell populations that imposed significant impact on the differences between HIV(+) AHCT recipients and controls are shown as violin plots for each time point (**Figures 5A–C**). Functions of cell subset are listed in **Table 2**.

**Figure 6:**
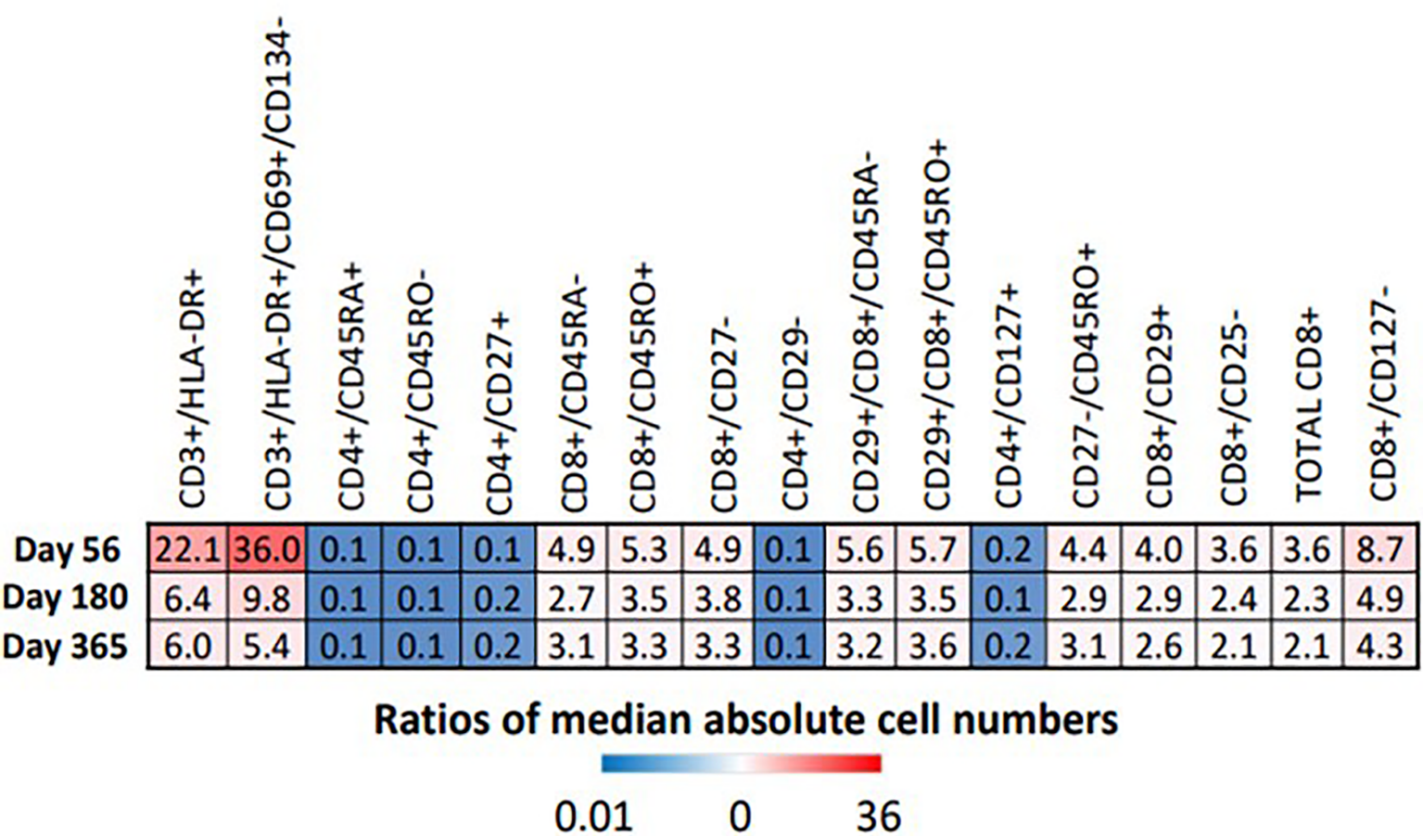
Heat map illustrates the fold difference between the absolute median number of immune cells in HIV(+) patients compared to controls, determined by Wilcoxon rank-sum test (p < 0.033).

Proportions and absolute numbers of each immune cell subset identified as significant by the FIS analysis were compared by Wilcoxon rank sum test with FDR correction. Heat maps representing the ratios of HIV(+) AHCT recipient median absolute cell numbers to healthy controls are displayed in **Figure 6**. Fold change shown within each square of the heat maps. Similarly, the ratios of median cell proportions are shown in supplemental **Figure S3**.

All marker combinations that were identified as significant by FIS analysis showed significant differences in comparisons of median absolute cell numbers and median cell proportions (p<0.0033). For each immune cell subset, fold change of both the median total cell numbers and median cell proportions was either stable or diminished over time, indicating increasing similarity between HIV(+) AHCT recipients and healthy controls during the post-transplant year. However, significant differences remained, as shown by the heat maps in **Figures 6** and **S3**. HIV(+) AHCT recipients had greater absolute numbers and higher proportions of total activated T cells (CD3+/DLA-DR+ and CD3+/HLA-DR+/CD69+/CD134-); higher total central and effector memory cells (CD27-/CD45RO+); higher total cytotoxic CD8+ T cells; higher memory cytotoxic T cells (CD8+/CD45RO+, CD8+/CD45RA-, CD8+/CD45RO+/CD29+ and CD8+/CD45RA-/CD29+); and higher cytotoxic effector T cells (CD8+/CD127- and CD8+/CD27-). CD4+ T helper cell subsets including naïve cells positive for CD45RA or CD127, or lacking CD45RO, as well as CD29-negative T helper cells, were lower in absolute numbers and proportions in HIV(+) AHCT recipients. These findings suggest that HIV(+) AHCT recipients reconstitute as pro-inflammatory immune phenotypes, that are similar to cancer-free HIV(+) subjects on cART.

### Differences Between Specific Immune Cell Subsets in HIV(+) AHCT Recipients, HIV(-) AHCT Recipients and Healthy Controls

**Figure 7:**
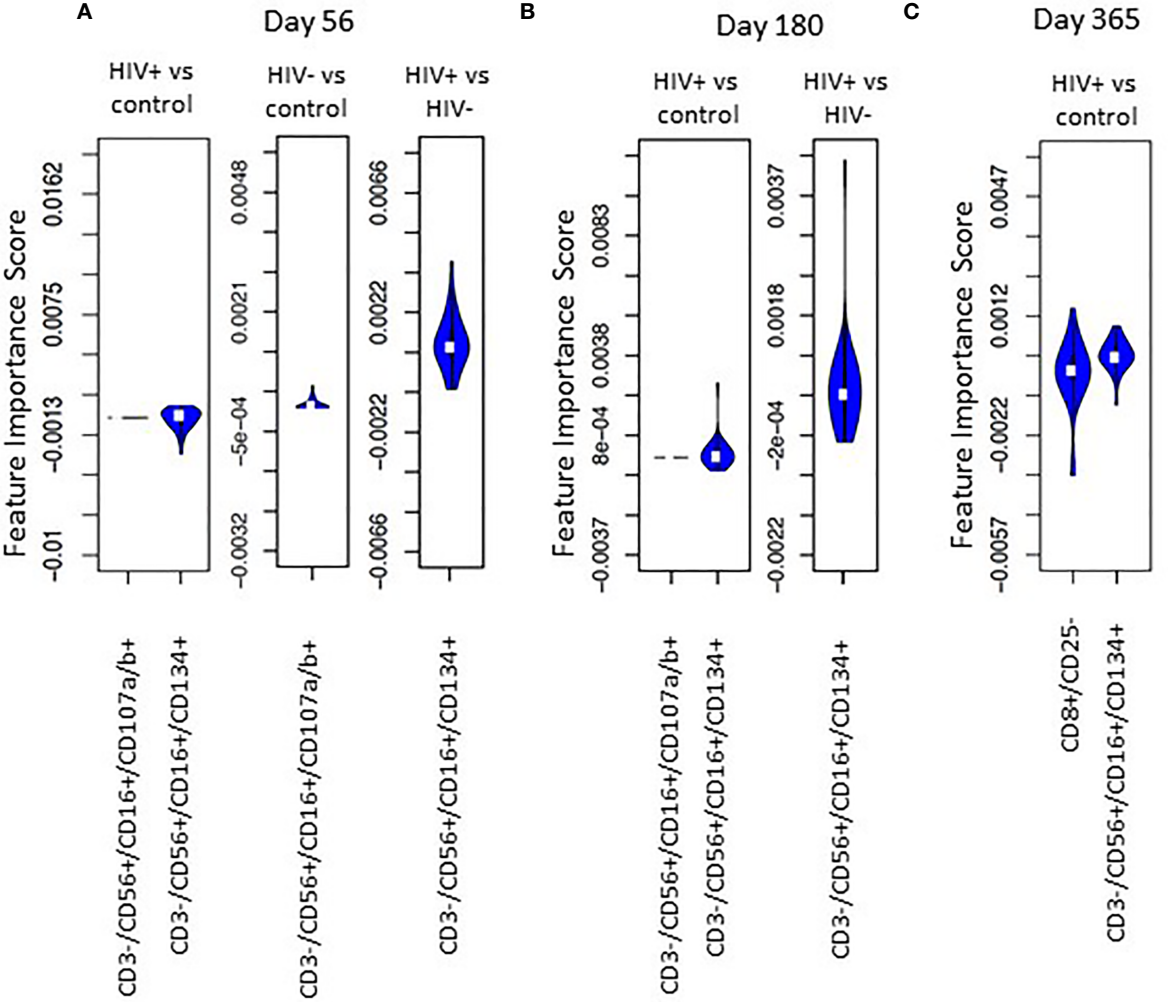
Violin plot shows the impact of specific immune subsets on the degree of difference between HIV(+), HIV(-) and control cohorts at days 56 **(A)**, 180 **(B)** and 365 **(C)** post-transplant.

**Figure 8:**
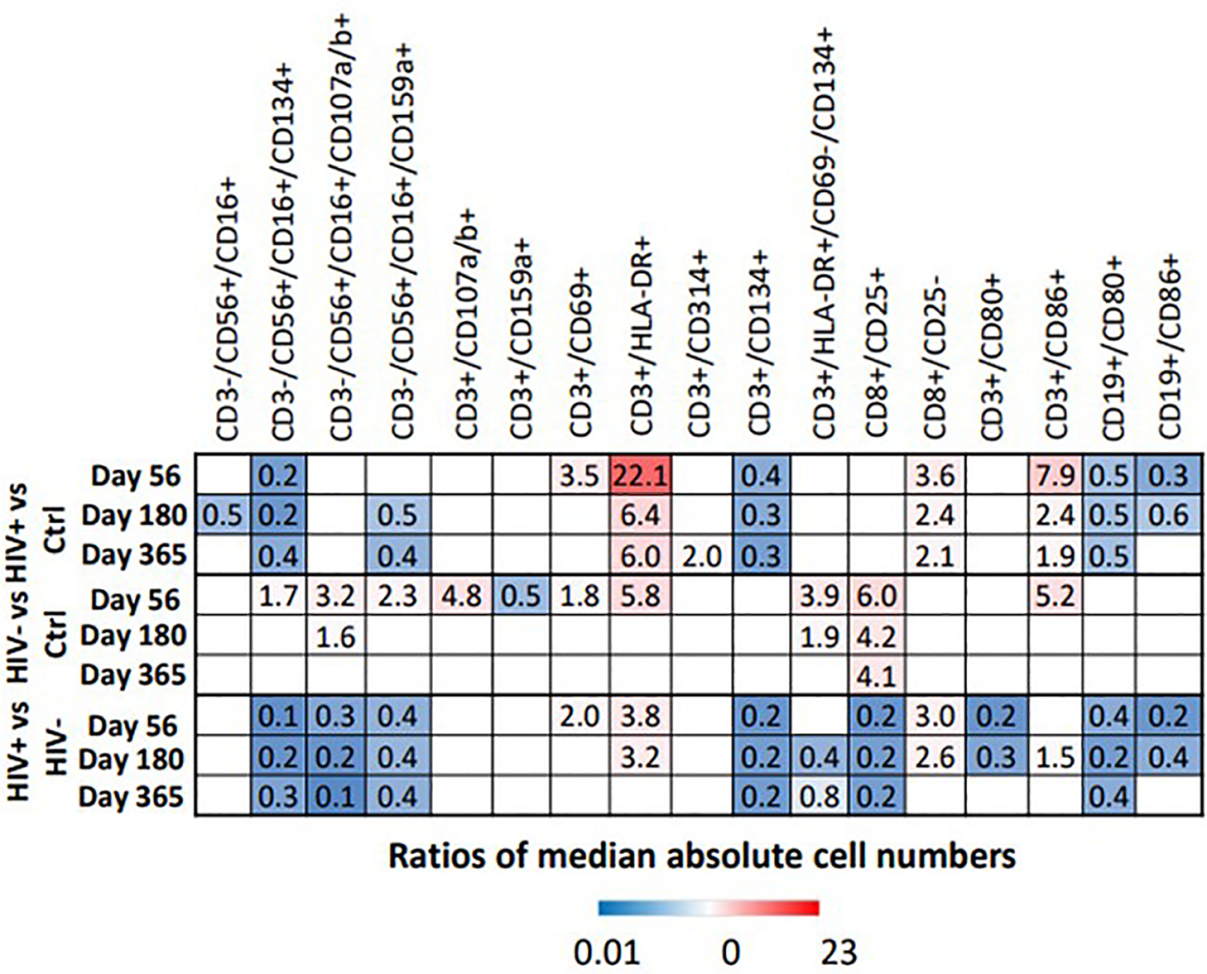
Heat map illustrates the fold difference between the absolute median number of immune cells in HIV(+) and HIV(-) patients and controls, determined by Wilcoxon rank-sum test (p < 0.006), while blank cells represent a lack of significant difference.

FIS analysis was applied to compare HIV(+) and HIV(-) AHCT recipient and healthy control cohorts across 18 immune cell populations at each time point (**Figures 7A–C**). Application of the analytic technique across a smaller total number of immune marker combinations increased the stringency of the analysis, which likely contributed to a smaller number of immune cell subsets that demonstrated a significant impact on the difference between cohort lymphocytes. Comparison of HIV(+) AHCT recipients and healthy controls revealed the significance of activated cytotoxic NK cells (CD3-/CD56+/CD16+/CD134+) at all time points (**Figure 7**), with absolute numbers significantly lower in HIV(+) AHCT recipients compared to HIV(-) AHCT recipients and to healthy controls as determined by Wicoxon rank sum test (**Figure 8** and **Table S2**). Degranulating cytotoxic NK cells (CD3-/CD56+/CD16+/CD107a/b+) were also significant in the FIS analysis as defining the differences between HIV(+) AHCT recipients and healthy controls at Days 56 and 180 post-AHCT (**Figure 7**). Absolute numbers of CD3-/CD56+/CD16+/CD107a/b+ cells were significantly lower in HIV(+) AHCT recipients compared to HIV(-) AHCT recipients at all time points by Wilcoxon rank sum test (**Figure 8** and **Table S2**), but no significant difference was found between HIV(+) AHCT recipients and healthy controls. Comparison of HIV(-) AHCT recipients and healthy controls also showed degranulating cytotoxic NK cells (CD3-/CD56+/CD16+/CD107a/b+) at Day 56 post-AHCT but not at subsequent time points, suggesting that innate immunity reconstitutes differently in HIV(+) and HIV(-) AHCT recipients. Absolute numbers were higher in HIV(-) AHCT recipients than in healthy controls on Days 56 and 180 by Wilcoxon rank sum test (**Figure 8** and **Table S2**), but significant differences resolved by Day 365 post-transplant. Furthermore, HIV(+) but not HIV(-) AHCT recipients showed cytotoxic T cells (CD8+/CD25-) as significant when compared to healthy controls at Day 365 post-AHCT. These findings reinforce our observation of the significant impact of HIV status on post-transplant cellular immune reconstitution: both HIV(+) and HIV(-) patients approach healthy controls over time, but significant differences between innate immune cells in the HIV(+) cohort and healthy control persist at 1 year post-HSCT. HIV(+) subjects demonstrate incomplete reconstitution of the activated and degranulating NK cell compartments as significantly different from healthy controls in the FIS analysis.

Independently of the FIS analysis, comparisons of HIV(+) AHCT recipients, HIV(-) AHCT recipients and healthy controls by Wilcoxon rank sum test with FDG correction identified multiple differences in the T, NK and B cell subsets, as summarized below (p<0.006, **Figure 8** and **Table S2**).

NK Cell Subsets: Multiple significant differences between absolute numbers of NK cell subsets were noted, as described below (p<0.006, **Figure 8** and **Table S2**). Absolute numbers of cytotoxic NK cells (CD3-/CD56+/CD16+) were significantly lower in HIV(+) AHCT recipients compared to healthy controls on Day 180. Absolute number of activated cytotoxic NK cells CD3-/CD56+/CD16+/CD134+ were lower in HIV(+) AHCT recipients compared to healthy controls and to HIV(-) AHCT recipients at all time points. HIV(-) AHCT recipients had a higher number of these cells compared to healthy controls at Day 56, but subsequently became similar to HC. Cytotoxic, degranulating NK cells (CD3-/CD56+/CD16+/CD107a/b+) were virtually undetectable in healthy controls and HIV(+) AHCT recipients, but detectable and significantly elevated in HIV(-) AHCT recipients compared to other cohorts on Days 56 and 180, although absolute numbers were low (**Table S2**). Furthermore, NK cells expressing the NKG2A inhibitory receptor (CD16+/CD56+/CD159a+) were lower in HIV(+) patients compared to healthy controls on Days 180 and 365, and higher in HIV(-) AHCT patients compared to healthy controls on Day 56 only. By Day 365, all NK cell subsets in HIV(-) AHCT recipients were not significantly different from those of healthy controls, while significant differences persisted in 2 NK cell subsets: CD3-/CD16+/CD56+/CD159a+ cells were lower in HIV(+) AHCT recipients compared to healthy controls, and CD3-/CD56+/CD16+/CD314+ cells that lower in HIV(+) AHCT recipients compared to HIV(-) AHCT recipients and healthy controls (**Figure 8** and **Table S2**). These findings indicate significant differences in the reconstitution of the NK cell compartment throughout the post-AHCT year in HIV(+) compared to HIV(-) AHCT recipients.

T Lymphocyte Subsets: At the initial post-AHCT time point (Day 56), both HIV(+) and HIV(-) AHCT recipients had higher absolute numbers of activated T cells (CD3+/HLA-DR+) compared to healthy controls (p<0.006, **Figure 6B** and **Table S2**). Significant differences in the CD3+/HLA-DR+ cell subset between HIV(-) AHCT recipients and healthy controls were no longer present on Day 180 and 365. Significant differences in absolute numbers of CD3+/HLA-DR+ between HIV(+) AHCT recipients and healthy controls persisted throughout the post-AHCT year. Furthermore, CD3+/HLA-DR+ cells remained significantly higher in HIV(+) AHCT recipients than in HIV(-) AHCT recipients at 56 and 180 days post-AHCT. A similar trend was observed in the absolute numbers of activated CD3+/CD86+ T cells. However, T cells expressing the late activation marker CD134 (CD3+/CD134+) were lower in HIV(+) patients. Activated T cells CD3+/CD134+/CD69-/HLA-DR+ were higher in HIV(-) patients than healthy controls at Day 56 and 180, but the difference resolved by Day 365; differences in this subset between HIV(+) patients and healthy controls were not significant. These findings are consistent with the features of increased T cell activation observed in individuals with chronic HIV infection controlled by cART, although the numbers of activated T cells decrease with initiation of antiviral therapy, and in individuals with undetectable HIV viral load without cART (22, 23). Our findings, in concert with previous reports, indicate that significant immune dysregulation persists in the HIV(+) setting despite undetectable viral load. Activated cytotoxic T cells (CD8+/CD25+) were elevated in HIV(-) patients compared to HIV(+) patients and healthy controls across all time points, but differences between HIV(+) and healthy controls were not significant. Overall, at Day 356, only CD8+/CD25+ activated cytotoxic T cells were different in HIV(-) patients compared to healthy controls, while differences remained significant across 5 subsets (activated T cells CD3+/CD134+, CD3+/HLA-DR+ and CD3+/CD314+, cytotoxic T cells CD8+/CD25- and effector memory T cells CD3+/CD86+) in HIV(+) patients compared to healthy controls. These results indicate appreciable differences in the T cell subset reconstitution in HIV(+) and HIV(-) AHCT recipients, with an emphasis on pro-inflammatory cell phenotypes present in the HIV(+) cohort.

B Lymphocyte Subsets: Activated B cells (CD19+/CD80+) were lower in HIV(+) AHCT recipients compared with healthy controls and with HIV(-) AHCT recipients at all time points (p<0.006, **Figure 6B** and **Table S2**). A comparable pattern was observed in activated B cells marked by CD19+/CD86+, although differences between all cohorts resolved by Day 365. In the HIV(-) cohort, activated B cells were not significantly different from healthy controls at all time points. We hypothesized that the reduction in B cells that was observed in HIV(+) AHCT recipients might have been attributed to B cell depleting therapy with agents such as rituximab that the HIV(+) patients may have received pre-AHCT for treatment of HIV-associated lymphoma, which would not have been administered to the HIV(-) AHCT recipient patient cohort that received treatment for multiple myeloma. Given the discordance between the two AHCT recipient cohorts, we performed an independent comparison of a sub-cohort consisting of HIV(+) patients that had Hodgkin lymphoma, and would not have been exposed to B cell depleting agents, to the HIV(-) AHCT cohort. Remarkably, the differences in activated B cells (CD19+/CD80+ and CD18+/CD86+) persisted when 14 HIV(+) AHCT recipients with Hodgkin lymphoma were compared directly to HIV(-) AHCT recipients by Wilcoxon rank sum analysis (p<0.0185, **Table S3**). These results indicate that decreased absolute numbers of B cells may be secondary to chronic HIV infection and not due to B cell-depleting treatment, which would have been administered to the CD20+ B cell lymphoma patients but not to the Hodgkin lymphoma patients. Furthermore, trends in the reconstitution of the NK cell and T cell compartments in the HIV(+) HL AHCT recipients mimicked those observed in the entire HIV(+) cohort (**Table S3**).

Overall, our findings showed that innate and adaptive cellular immune reconstitution occur at different rates and along seprate trajectories in HIV(+) AHCT recipients and in HIV(-) AHCT recipients. Factors including chronic, albeit controlled HIV infection, use of cART, different modes of pre-transplant myeloablative chemotherapy conditioning (BEAM in the HIV(+) cohort and melphalan in the HIV(-) cohort) and different hematologic malignancy history may contribute toward the differences that we observed. A detailed assessment of phenotypic and functional NK cell subset reconstitution in HIV(-) AHCT recipients after BEAM conditioning has not been previously performed. Further studies of NK cell populations in HIV(-) recipients of AHCT for treatment of hematologic malignancies, and in HIV(+) individuals on cART that have not undergone AHCT, are needed to distinguish between these possible reasons.

### Functional T Cell Recovery in HIV(+) Patients After AHCT

**Figure 9:**
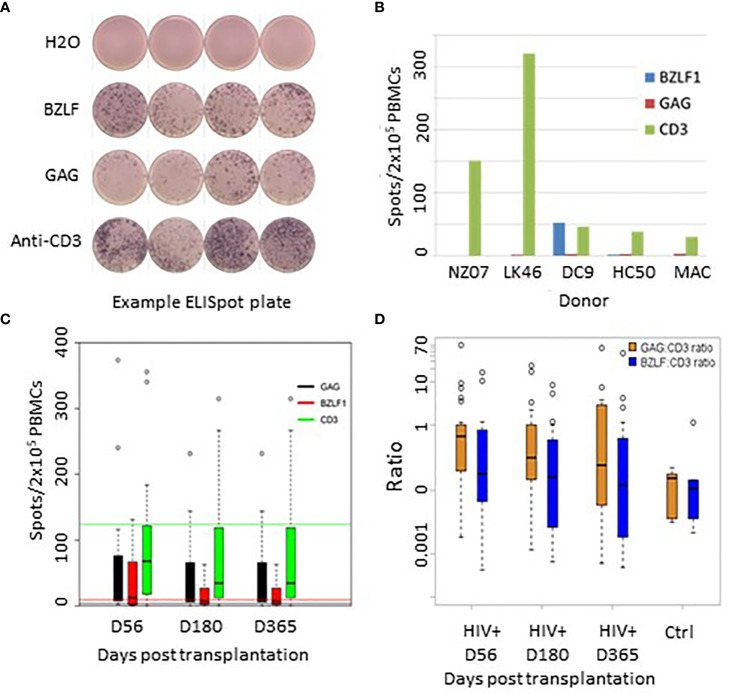
**(A)** Representative Elispot plate raw data. **(B)** Recall antigen responses in 5 healthy EBV+/HIV- donors. **(C)** Recall responses to GAG, BZLF, and anti TCR post transplant. Green line = TCR mean response from healthy donors; Red line = BZLF1 mean responses from healthy controls; Black line = background GAG responses from healthy controls. **(D)** Recall response ratios controlled for responsiveness to TCR stimulation with CD3-directed antibodies.

Functional immune recovery was evaluated by IFNγFELISpot assay on PBMCs that were collected on Days 56, 180, and 360 post-AHCT and stimulated with pepmixes derived from the EBV recall antigen BZLF1 or HIV recall antigen GAG. For positive control, PBMCs were stimulated with CD3-directed antibody (**Figure 9**).

Of 30 evaluated HIV(+) patients, 28 patients demonstrated measurable IFNγFproduction in response to the HIV GAG pepmix (spots/2x10^5^ PBMC, range: 8-615), 21 showed measurable response to the EBV BZLF1 pepmix (range 12-450), and all patients demonstrated responsiveness to CD3-directed stimulation.

An exemplary readout of the ELISpot assay is shown in **Figure 9A**. **Figure 9B** shows baseline levels of IFNγFproduction in healthy controls. **Figure 9C** shows average absolute recall antigen responses in HIV(+) patients at each time point. HIV(+) AHCT recipient responses are shown as vertical bars, while horizontal lines of the corresponding colors represent aggregate mean corresponding responses from healthy donors. To assess the degree of specific T cell responsiveness to recall antigens in relation to overall T cell activation, absolute response values to recall antigens were normalized to overall T cell activation with CD3-directed antibodies (**Figure 9D**). These results indicate a positive functional recovery of adaptive T lymphocytes’ ability to respond to TCR stimulation and to generate memory-specific responses to viral recall antigens over time. HIV(+) patients response to GAG, normalized to TCR stimulation, showed higher IFNγFproduction as compared to PBMCs from HIV(-) healthy volunteer controls on Days 56 and 180 (p-values 0.017 and 0.04, respectively) and higher but not significantly different at Day 365 (p-value 0.22). There were no significant differences between HIV(+) AHCT recipient and healthy control responses to BZLF1 (p-values 0.55, 0.71 and 0.88 for comparisons at Days 56, 180 and 365).

## Discussion

We report a first in-depth, comprehensive flow cytometry-based and functional comparison of immune reconstitution in HIV(+) and HIV(-) recipients of autologous stem cell transplantation. Within the first year following AHCT, we observed persistently lower absolute numbers of CD4+ helper T cell subsets, higher numbers of CD8+ T cell subsets and activated T cells, lower numbers of activated B cells and lower numbers of subsets of natural killer (NK) cell subsets expressing markers of activation and inhibition in HIV(+) AHCT recipients, compared to HIV(-) AHCT recipients and healthy controls. Remarkably, pro-inflammatory immune features associated with chronic, controlled HIV infection persisted in HIV(+) AHCT recipients despite multiple rounds of chemo-immunotherapy that included myeloablative chemotherapy. T lymphocytes from HIV(+) patients responded robustly to recall antigen challenge with HIV and Epstein-Barr virus (EBV) pepmixes. Overall, these data indicate a trend toward an activation-prone state of the adaptive immune cells in HIV(+) patients after AHCT that is maintained despite undetectable HIV viral load in the majority of patients, and despite response of their lymphoma to myeloablative therapy. HIV(-) AHCT recipients, on the other hand, demonstrated resolution of multiple features of immune activation after the post-transplant year, and acquired greater similarity to healthy controls. We conclude that patients with HIV-related lymphoma reconstitute immune features of chronic HIV infection post-transplant despite control of viremia.

Limitations of our study include the lack of pre-transplant immune profiling of our subjects and the lack of matching of the HIV(+) and HIV(-) AHCT recipient cohorts by hematologic malignancy and conditioning chemotherapy. These differences may appear as potential confounders. We did not have access to samples from HIV(-) AHCT recipients with aggressive lymphoma. Furthermore, we have not been able to find similarly extensive immune reconstitution studies of HIV(-) lymphoma patients in the literature, although studies describing immune reconstitution with a lesser degree of detail exist. This highlights the importance of ours and similar work approaching in-depth immune reconstitution analyses of various AHCT recipient populations. Publication of these studies would enhance understanding of the impact of conditioning regimens and inflammatory triggers on long term post-auto transplant immune reconstitution, which, as we see from our study, can follow diverse avenues over the post-transplant year.

Prior analysis of the kinetics of T, NK and B cell recovery in HIV(+) and HIV(-) patients has shown that, while CD4+ T cell recovery lagged by 3 months in HIV(+) patients, there was no difference in the timing of recovery in CD8+ T cells, CD56+ NK cell and CD19+ B cell (16). The study did not investigate reconstitution of multiple immune cell subsets, such as activated or degranulating T cell subsets. HIV(+) AHCT recipients also showed similar dynamics of recovery of thymopoiesis as HIV(-) counterparts, as measured by signal joint T cell receptor excision circles (16). An overall higher CD8+ T cell and an inverted ratio of CD4+ cells to CD8+ cells in HIV(+) AHCT recipients was also noted in this study (16). The return of the HIV (–) immune features to match healthy controls is consistent with a previously published report demonstrating recovery of NK cells in 3 months and T cells in 6 months after AHCT in HIV(-) patients (24).

Immune features that persisted in HIV(+) AHCT recipients (distinct from HIV(-) AHCT and healthy controls) were consistent with previously described activation-prone changes observed in chronic HIV infection treated with cART (25–27). Chronic, treated HIV infection is associated with immune dysregulation, despite an effective control of viremia and improvement in CD4 counts. Hallmarks of chronic HIV infection include elevated CD8+ T cell counts and elevated expression of activation markers such as HLA-DR (28, 29). HIV(+) patients with chronic infection treated with cART demonstrate lower absolute CD4+ and higher CD8+ T cell counts, increased levels of immune activation as marked by soluble CD14 level and increased percentages of CD38+/HLA-DR+ expression in both CD4+ and CD8+ T cells, and higher percentage of CD4+ T cells expressing markers of senescence, compared to age-matched controls (30, 31). Depletion of naïve CD4+ T cells in patients on long-term cART was also reported (31, 32). We have observed many attributes seen in the T cell compartment of patients with chronic, controlled HIV infection in HIV(+) AHCT recipients. These included an increase in the absolute numbers and proportions of activated T cell subsets, increase in memory T cell subsets and a reduction in naïve T cell subsets in HIV(+) AHCT recipients, compared to HIV(-) counterparts and healthy controls. Interestingly, activated T cells expressing CD134 turned out to be lower in HIV(+) patients compared to HIV(-) patients and healthy controls. CD134 is a marker of late T cell activation that has been implicated in enhanced T cell proliferation, survival, promotion of memory and effector T cell populations and tumor-directed responses (33–38). CD134 was also reported to enhance cytotoxic T cell responses to HIV, EBV and influenza (39). Diminished CD134 expression in HIV(+) individuals is a novel finding that, to our knowledge, has not been previously reported. Diminished CD134 expression might imply difficulties in mounting effective memory immune responses in HIV(+) patients post-AHCT, and contribute to the immune features that play a role in increased susceptibility of individuals with chronic, controlled HIV infection to malignancies related to oncogenic viral infections. Although we observed robust total T cell responses to EBV in HIV(+) AHCT recipients by ELISpot, further studies are warranted to investigate virus responsiveness in specific sub-sets of cytotoxic and helper T cells.

We have observed no difference in total NK cell numbers when comparing HIV(+) and HIV(-) AHCT recipients at 1 year post-transplant and healthy controls. However, cytotoxic NK cell populations bearing functional markers of activation and inhibition were significantly reduced in the HIV(+) cohort, while being increased or similar to control in the HIV(-) AHCT recipient cohort. Subsets of interest included NK cells expressing CD107a, NKG2A (up to Day 180), CD159a and CD314 (throughout the post-transplant year). Interestingly, previously published studies have generally demonstrated features of increased NK cell activation and impaired NK cell function in viremic HIV(+) individuals, with partial restoration of NK cell function upon initiation of cART and administration of IL-2 treatment (40). A fall in the numbers of NK cell expressing the inhibitory receptor NKG2A in viremic HIV(+) patients, and restoration of expression in patients on cART, have been reported (41). Numbers of regulatory CD56+/CD16+/- NK cells were reported as unchanged in HIV(+) patients, both treated and untreated with cART (42). Patients in our HIV(+) AHCT recipient cohort were receiving cART throughout the post-transplant period, except for a planned interruption of a median 15.5 days around the time of AHCT, and by day 365, 82.6% of the patients had an undetectable HIV viral load (17). Rapid NK cell reconstitution and restoration of absolute cell numbers to the normal donor range has been reported in HIV(-) recipients of AHCT for treatment of plasma cell myeloma (43). A linear NK cell maturation model suggests that CD16+ NK cell subsets represent mature NK cell phenotypes (44, 45). NK cell maturation studies have shown that NK cells derived from umbilical cord blood express less degranulation marker CD107a and show less antibody-dependent cellular cytotoxicity, compared to adult-derived NK cells (46). A greater fraction of umbilical cord NK cells also expressed markers of immaturity, such as CD159a (NKG2A) (46). Our observation of higher numbers of CD3-/CD16+/CD56+/CD159a+ cells in HIV(-) AHCT recipients at earlier post-transplant time points, and lower numbers of activated and degranulating cytotoxic NK cells in the HIV(+) AHCT recipient cohort throughout the post-transplant year, suggest that NK cell maturation occurred at different rates in the HIV(+) and HIV(-) AHCT recipients. Innate immune cell subset maturation and/or response to acute infection may be impaired in the HIV(+) AHCT recipient cohort suggesting impaired NK cell reconstitution and possibly impaired NK cell subset maturation follows myeloablative chemotherapy and AHCT for treatment of aggressive relapsed/refractory lymphoma in the presence of controlled HIV infection.

Likewise, we observed diminished B lymphocyte recovery in HIV(+) patients undergoing AHCT compared to HIV(-) AHCT recipients and healthy control subjects. Reduced B cell proliferative responses and increased B cell apoptosis have been reported in individuals with HIV viremia (47, 48). Reduced survival of memory B cells has also been reported in patients with chronic, controlled HIV infection (49). Our data describing reconstitution of B cell subsets in HIV(+) AHCT appear to be consistent with observations in patients with chronic HIV infection treated with cART. Importantly, the activated B cell depletion seen in our HIV(+) cohort appeared to be independent of these patients receiving rituximab, since similar loss of B cells was seen in HIV(+) HL patients compared to the HIV(-) AHCT cohort. Hypergammaglobulinemia has been reported in HIV(+) individuals, and was reported to correct with initiation of cART (50). This concept appears to be consistent with our observation of reduced numbers of activated circulating B cells in HIV(+) AHCT recipients, although data on circulating immunoglobulin levels were not collected systematically in our patient cohorts.

We found that HIV(+) transplant recipients possess T cells capable of producing INFγ upon stimulation with EBV BZLF1*-*derived peptides. Responses to BZLF1 pepmix and CD3-specific antibodies were equivalent between HIV(+) AHCT recipients and healthy controls at all time points. It has been previously demonstrated that viral-specific T cell responses are initially diminished after allogeneic stem cell or double cord blood transplantation in HIV(-) patients (51–53). This is the first study to our knowledge that describes the longitudinal viral antigen-specific T cell activity among HIV(+) recipients of autologous stem cell transplantation. The ability to maintain active virus-specific memory responses likely contributed to the low transplant-related mortality (5.2%) of HIV(+) AHCT recipients, which was not significantly different from HIV(-) historical controls (17).

In-depth flow-cytometry based analysis of immune recovery showed that HIV(+) AHCT recipients demonstrate patterns of lymphocyte profiling consistent with that seen in patients with chronic HIV infection who are treated with cART. Our analysis did not identify a correlation between infection rates and immune reconstitution trends. Thus, when taken in the context of positive clinical outcomes of HIV(+) patients described by Alvarnas et al. (17), the distinct pro-inflammatory features of immune reconstitution described here provide justification for additional longitudinal studies in this unique patient population.

## Data Availability Statement

The original contributions presented in the study are included in the article/**Supplementary Material**. Further inquiries can be directed to the corresponding authors.

## Ethics Statement

This is a correlative study that used de-identified samples from patients enrolled on the BMT CTN 0803/AMC 071/NCT01141712 and NCT00569309 trials. Multiple institutions recruited patients onto the BMT CTN 0803/AMC 071/NCT01141712 trial. In each institution, patient enrollment was approved by the Institutional Review Board in accordance to the ethical principles regarding human experimentation stated in the Declaration of Helsinki. Written informed consent was received at each individual institution from participants prior to inclusion in the study. De-identified blood samples were shipped to the Ohio State University for analysis that incorporated secondary use of de-identified data and did not constitute human subjects research. The Ohio State University recruited patients onto the NCT00569309 trial, and use of de-identified data was approved by the Ohio State University Institutional Review Board (Study Number: 2021C0016). Normal ranges for cell subsets were established by the clinical pathology laboratory using de-identified samples from normal donors according to clinical laboratory regulations. The patients/participants provided their written informed consent to participate in this study.

## Author Contributions

PS wrote the manuscript, analyzed data, interpreted data analysis, created figures. MP, MS, EM, XZ, MM, and AM analyzed data. EHA, SS, RP, and RK conducted experiments. AM created figures and edited manuscript. JL-R and RL designed research studies. GA, ErA, SD, LK, AN, UP, LM, AK, WW, NW, NS, CH, SF, and WN acquired data. SD, JA, RA, and GL designed research studies and acquired data. RB designed research and wrote the manuscript. All authors contributed to the article and approved the submitted version.

## Funding

Funding for this Blood and Marrow Transplant Clinical Trials Network (BMT CTN) and AIDS Malignancy Consortium (AMC) study was provided by HHSN261200622012C-009 from the National Cancer Institute (NCI) and by the AIDS Malignancy Consortium (AMC) through NCI grant U01CA121947. The BMT CTN infrastructure is supported in part by grant U10HL069294 to the Blood and Marrow Transplant Clinical Trials Network from the National Heart, Lung, and Blood Institute and NCI. PS has received funding through an AMC Translational Fellowship funded by the AMC through the grant U01CA121947 in 2017-2019 and T32 fellowship through the grant T32 CA009338 from the NIH. Research reported in this publication was also supported by The Ohio State University Comprehensive Cancer Center and the National Institutes of Health under grant number P30 CA016058. The study was coordinated by the AIDS Malignancy Consortium and supported in part by Public Health Service Grant No. UM1 CA121947 and the National Cancer Institute, National Institutes of Health, and the Department of Health and Human Services. EHA is supported by NIH-T32 grant T32CA090223.

## Author Disclaimer

The content is solely the responsibility of the authors and does not necessarily represent the official views of the National Institutes of Health.

## Conflict of Interest

PS, Seattle Genetics, Research funding. AK, Celgene, Consultancy, Speakers Bureau, Millennium/Takeda, Consultancy, Speakers Bureau, Onyx, Consultancy, Speakers Bureau, Janssen, Consultancy, Speakers Bureau. Hofmeister, Signal Genetics, Inc., Membership on an entity’s Board of Directors or advisory committees, Celgene, Research Funding; Arno Therapeutics, Inc., Research Funding, Incyte, Corp, Membership on an entity’s Board of Directors or advisory committees, Janssen, Pharmaceutical Companies of Johnson & Johnson, Research Funding, Karyopharm Therapeutics, Research Funding, Takeda Pharmaceutical Company, Research Funding, Teva, Membership on an entity’s Board of Directors or advisory committees. SF, Mustang Therapeutics, Other, Construct licensed by City of Hope. WN, Atara Biotherapeutics, employment, stock ownership. GL, Boehringer Ingelheim, Research Funding, Beckman Coulter, Research Funding, Stemline Therapeutics Inc., Research Funding, Genentech. RB, Prelude Therapeutics, Research Funding and Scientific Advisory Board, Viracta, Scientific Advisory Board. AN, Janssen, Membership on a Board or Advisory Committee; Medscape, Honoraria; Morphosys, Membership on a Board or Advisory Committee; Pharmacyclics, Research Funding, Honoraria; Rafael Pharma, Research Funding; Epizyme, Membership on a Board or Advisory Committee; Physician Education Resource, Consulting. MM and AMM are employed by EMMES Company.

The remaining authors declare that the research was conducted in the absence of any commercial or financial relationships that could be construed as a potential conflict of interest.

## Publisher’s Note

All claims expressed in this article are solely those of the authors and do not necessarily represent those of their affiliated organizations, or those of the publisher, the editors and the reviewers. Any product that may be evaluated in this article, or claim that may be made by its manufacturer, is not guaranteed or endorsed by the publisher.
