# Supplementary material for: Immune Recovery Following Autologous Hematopoietic Stem Cell Transplantation in HIV-Related Lymphoma Patients on the BMT CTN 0803/AMC 071 Trial
Source: Front Immunol. 2021 Sep 3;12:700045. doi: 10.3389/fimmu.2021.700045 (PMC8446430; doi:10.3389/fimmu.2021.700045)
Supplement: Supplementary file 1 [file DataSheet_1.docx]

| **Function** | **Biomarker combination** | **Compare** | **Day** | **p value** | **Median** | | **Higher count** |
| --- | --- | --- | --- | --- | --- | --- | --- |
|  |  |  |  |  | **HIV(+)** | **HC** |  |
| Total B cells | B cells CD19+ | HIV vs. control | 56 | <0.0001 | 1.8 | 248 | control |
|  | B cells CD19+ | HIV vs. control | 180 | 0.0005 | 138.6 | 248 | control |
| Activated B cells | B cells CD19+/CD80+ | HIV vs. control | 56 | <0.0001 | 0 | 2 | control |
|  | B cells CD19+/CD80+ | HIV vs. control | 180 | 0.0001 | 0 | 2 | control |
|  | B cells CD19+/CD80+ | HIV vs. control | 365 | 0.0002 | 0 | 2 | control |
|  | B cells CD19+/CD86+ | HIV vs. control | 56 | <0.0001 | 0 | 3 | control |
|  | B cells CD19+/CD86+ | HIV vs. control | 180 | 0.001 | 1.7 | 3 | control |
| Total T cells | T cells CD3+/CD56-/CD16- | HIV vs. control | 56 | 0.0011 | 1943 | 1470 | HIV |
| Activated T cells | T cells CD16-/CD56-/CD3+/CD117- | HIV vs. control | 56 | 0.0027 | 1925 | 1418 | HIV |
|  | T cells CD69+/CD3+ | HIV vs. control | 56 | <0.0001 | 85 | 24 | HIV |
|  | T cells CD134+/CD3+ | HIV vs. control | 56 | <0.0001 | 7 | 20 | control |
|  | T cells CD134+/CD3+ | HIV vs. control | 180 | 0.0006 | 6.8 | 20 | control |
|  | T cells CD134+/CD3+ | HIV vs. control | 365 |  | 5.2 | 20 | control |
|  | T cells CD3+/HLA-DR+ | HIV vs. control | 56 | <0.0001 | 751.2 | 34 | HIV |
|  | T cells CD3+/HLA-DR+ | HIV vs. control | 180 | <0.0001 | 216.2 | 34 | HIV |
|  | T cells CD3+/HLA-DR+ | HIV vs. control | 365 | <0.0001 | 202.5 | 34 | HIV |
|  | T cells HLA-DR-/CD69+/CD3+/CD134+ | HIV vs. control | 56 | 0.0029 | 0 | 0 | control |
|  | T cells HLA-DR+/CD69+/CD3+/CD134- | HIV vs. control | 56 | <0.0001 | 36 | 0 | HIV |
|  | T cells HLA-DR+/CD69+/CD3+/CD134- | HIV vs. control | 180 | <0.0001 | 9.8 | 0 | HIV |
|  | T cells HLA-DR+/CD69+/CD3+/CD134- | HIV vs. control | 365 | <0.0001 | 5.4 | 0 | HIV |
|  | T cells CD3+/CD86+ | HIV vs. control | 56 | <0.0001 | 31.5 | 4 | HIV |
|  | T cells CD3+/CD86+ | HIV vs. control | 180 | <0.0001 | 9.4 | 4 | HIV |
|  | T cells CD3+/CD86+ | HIV vs. control | 365 | 0.0003 | 7.5 | 4 | HIV |
|  | T cells CD3+/CD314+ | HIV vs. control | 365 | 0.0002 | 846 | 426 | HIV |
|  | T cells CD3+/CD158b+ | HIV vs. control | 56 | <0.0001 | 64.8 | 19 | HIV |
|  | T cells CD3+/CD158b+ | HIV vs. control | 365 | 0.0013 | 53.9 | 19 | HIV |
|  | T cells CD3+/CD69+/CD158b+ | HIV vs. control | 56 | 0.0002 | 1.7 | 0 | HIV |
| Naïve and memory T cells | Total CD45RA+/CD45RO- | HIV vs. control | 180 | 0.0007 | 780 | 1043 | control |
|  | Total CD45RA-/CD45RO+ | HIV vs. control | 56 | 0.0003 | 1160 | 818 | HIV |
|  | Total CD45RA+/CD45RO+ | HIV vs. control | 56 | <0.0001 | 338 | 109 | HIV |
|  | Total CD45RA+/CD45RO+ | HIV vs. control | 180 | <0.0001 | 224 | 109 | HIV |
|  | Total CD45RA+/CD45RO+ | HIV vs. control | 365 | 0.0001 | 261.1 | 109 | HIV |
|  | Total CD27+/CD45RA+ | HIV vs. control | 56 | <0.0001 | 270.6 | 698 | control |
|  | Total CD27+/CD45RA+ | HIV vs. control | 180 | <0.0001 | 255.5 | 698 | control |
|  | Total CD27+/CD45RA+ | HIV vs. control | 365 | <0.0001 | 286.1 | 698 | control |
|  | Total CD27+/CD45RO+ | HIV vs. control | 180 | 0.003 | 505.4 | 791 | control |
|  | Total CD27-/CD45RO+ | HIV vs. control | 56 | <0.0001 | 588 | 133 | HIV |
|  | Total CD27-/CD45RO+ | HIV vs. control | 180 | 0.0001 | 384.2 | 133 | HIV |
|  | Total CD27-/CD45RO+ | HIV vs. control | 365 | 0.0001 | 406 | 133 | HIV |
|  | Total CD29+/CD45RA+/CD45RO- | HIV vs. control | 180 | 0.0018 | 789.6 | 1066 | control |
|  | Total CD29+/CD45RA-/CD45RO+ | HIV vs. control | 56 | 0.0005 | 1139 | 824 | HIV |
|  | Total CD29+/CD45RA+/CD45RO+ | HIV vs. control | 56 | <0.0001 | 304 | 96 | HIV |
|  | Total CD29+/CD45RA+/CD45RO+ | HIV vs. control | 365 | 0.0005 | 216 | 96 | HIV |
|  | Total CD29+/CD45RO+ | HIV vs. control | 56 | <0.0001 | 1281 | 722 | HIV |
|  | Total CD29-/CD45RO+ | HIV vs. control | 56 | <0.0001 | 89.9 | 187 | control |
|  | Total CD29-/CD45RO+ | HIV vs. control | 180 | <0.0001 | 76.8 | 187 | control |
|  | Total CD29-/CD45RO+ | HIV vs. control | 365 | <0.0001 | 66.3 | 187 | control |
|  | Total CD29+/CD45RA+ | HIV vs. control | 56 | <0.0001 | 934.2 | 559 | HIV |
|  | Total CD29+/CD45RA+ | HIV vs. control | 180 | 0.0005 | 834.6 | 559 | HIV |
|  | Total CD29-/CD45RA+ | HIV vs. control | 56 | <0.0001 | 113.4 | 642 | control |
|  | Total CD29-/CD45RA+ | HIV vs. control | 180 | <0.0001 | 158.4 | 642 | control |
|  | Total CD29-/CD45RA+ | HIV vs. control | 365 | <0.0001 | 238 | 642 | control |
| Total T helper cells | Total CD4+ | HIV vs. control | 56 | <0.0001 | 235 | 992 | control |
|  | Total CD4+ | HIV vs. control | 180 | <0.0001 | 201 | 992 | control |
|  | Total CD4+ | HIV vs. control | 365 | <0.0001 | 280 | 992 | control |
| Naïve and memory T helper cells | T Cells CD4+/CD45RA+ | HIV vs. control | 56 | <0.0001 | 27 | 379 | control |
|  | T Cells CD4+/CD45RA+ | HIV vs. control | 180 | <0.0001 | 30.6 | 379 | control |
|  | T Cells CD4+/CD45RA+ | HIV vs. control | 365 | <0.0001 | 33 | 379 | control |
|  | T Cells CD4+/CD45RA- | HIV vs. control | 56 | <0.0001 | 202 | 602 | control |
|  | T Cells CD4+/CD45RA- | HIV vs. control | 180 | <0.0001 | 176.8 | 602 | control |
|  | T Cells CD4+/CD45RA- | HIV vs. control | 365 | <0.0001 | 215 | 602 | control |
|  | T Cells CD4+/CD45RO+ | HIV vs. control | 56 | <0.0001 | 208.8 | 614 | control |
|  | T Cells CD4+/CD45RO+ | HIV vs. control | 180 | <0.0001 | 176.8 | 614 | control |
|  | T Cells CD4+/CD45RO+ | HIV vs. control | 365 | <0.0001 | 211.2 | 614 | control |
|  | T Cells CD4+/CD45RO- | HIV vs. control | 56 | <0.0001 | 20 | 338 | control |
|  | T Cells CD4+/CD45RO- | HIV vs. control | 180 | <0.0001 | 26.4 | 338 | control |
|  | T Cells CD4+/CD45RO- | HIV vs. control | 365 | <0.0001 | 36 | 338 | control |
|  | T cells CD4+/CD27+ | HIV vs. control | 56 | <0.0001 | 113.6 | 876 | control |
|  | T cells CD4+/CD27+ | HIV vs. control | 180 | <0.0001 | 139.2 | 876 | control |
|  | T cells CD4+/CD27+ | HIV vs. control | 365 | <0.0001 | 160.2 | 876 | control |
|  | T cells CD4+/CD29+ | HIV vs. control | 56 | <0.0001 | 199.8 | 538 | control |
|  | T cells CD4+/CD29+ | HIV vs. control | 180 | <0.0001 | 168 | 538 | control |
|  | T cells CD4+/CD29+ | HIV vs. control | 365 | <0.0001 | 201.6 | 538 | control |
|  | T cells CD4+/CD29- | HIV vs. control | 56 | <0.0001 | 25.2 | 415 | control |
|  | T cells CD4+/CD29- | HIV vs. control | 180 | <0.0001 | 40 | 415 | control |
|  | T cells CD4+/CD29- | HIV vs. control | 365 | <0.0001 | 46.4 | 415 | control |
|  | T cells CD4+/CD127+ | HIV vs. control | 56 | <0.0001 | 144 | 892 | control |
|  | T cells CD4+/CD127+ | HIV vs. control | 180 | <0.0001 | 132 | 892 | control |
|  | T cells CD4+/CD127+ | HIV vs. control | 365 | <0.0001 | 173.8 | 892 | control |
| Th1 cells | T cells CD4+/CD183+ | HIV vs. control | 56 | <0.0001 | 144 | 365 | control |
|  | T cells CD4+/CD183+ | HIV vs. control | 180 | <0.0001 | 127.2 | 365 | control |
|  | T cells CD4+/CD183+ | HIV vs. control | 365 | <0.0001 | 153 | 365 | control |
| Th2 cells | T cells CD4+/CD194+ | HIV vs. control | 56 | <0.0001 | 28.8 | 128 | control |
|  | T cells CD4+/CD194+ | HIV vs. control | 180 | <0.0001 | 23.4 | 128 | control |
|  | T cells CD4+/CD194+ | HIV vs. control | 365 | 0.0003 | 41.6 | 128 | control |
| Treg | T cells CD8-/CD127-/CD4+/CD25+ | HIV vs. control | 56 | <0.0001 | 8.8 | 28 | control |
|  | T cells CD8-/CD127-/CD4+/CD25+ | HIV vs. control | 180 | <0.0001 | 4.6 | 28 | control |
|  | T cells CD8-/CD127-/CD4+/CD25+ | HIV vs. control | 365 | <0.0001 | 2.6 | 28 | control |
| Total cytotoxic T cells | Total CD8+ | HIV vs. control | 56 | <0.0001 | 1738 | 488 | HIV |
|  | Total CD8+ | HIV vs. control | 180 | <0.0001 | 1141 | 488 | HIV |
|  | Total CD8+ | HIV vs. control | 365 | <0.0001 | 1010 | 488 | HIV |
| Naïve and memory cytotoxic T cell subsets | T cells CD8+/CD45RA+ | HIV vs. control | 56 | <0.0001 | 899.2 | 399 | HIV |
|  | T cells CD8+/CD45RA+ | HIV vs. control | 180 | <0.0001 | 635.8 | 399 | HIV |
|  | T cells CD8+/CD45RA+ | HIV vs. control | 365 | 0,0025 | 546 | 399 | HIV |
|  | T cells CD8+/CD45RA- | HIV vs. control | 56 | <0.0001 | 927 | 173 | HIV |
|  | T cells CD8+/CD45RA- | HIV vs. control | 180 | <0.0001 | 468 | 173 | HIV |
|  | T cells CD8+/CD45RA- | HIV vs. control | 365 | <0.0001 | 535.6 | 173 | HIV |
|  | T cells CD8+/CD45RO+ | HIV vs. control | 56 | <0.0001 | 1166 | 221 | HIV |
|  | T cells CD8+/CD45RO+ | HIV vs. control | 180 | <0.0001 | 766.5 | 221 | HIV |
|  | T cells CD8+/CD45RO+ | HIV vs. control | 365 | <0.0001 | 722.8 | 221 | HIV |
|  | T cells CD8+/CD45RO- | HIV vs. control | 56 | 0.0001 | 591.8 | 339 | HIV |
|  | T cells CD8+/CD27+ | HIV vs. control | 56 | <0.0001 | 824 | 392 | HIV |
|  | T cells CD8+/CD27+ | HIV vs. control | 180 | 0.0016 | 525 | 392 | HIV |
|  | T cells CD8+/CD27- | HIV vs. control | 56 | <0.0001 | 864 | 175 | HIV |
|  | T cells CD8+/CD27- | HIV vs. control | 180 | <0.0001 | 669.6 | 175 | HIV |
|  | T cells CD8+/CD27- | HIV vs. control | 365 | <0.0001 | 570.7 | 175 | HIV |
|  | T cells CD29+/CD8+/CD45RA+ | HIV vs. control | 56 | <0.0001 | 886.4 | 406 | HIV |
|  | T cells CD29+/CD8+/CD45RA+ | HIV vs. control | 180 | 0.0001 | 622.7 | 406 | HIV |
|  | T cells CD29+/CD8+/CD45RA+ | HIV vs. control | 365 | 0.0024 | 528 | 406 | HIV |
|  | T cells CD29+/CD8+/CD45RA- | HIV vs. control | 56 | <0.0001 | 947.1 | 170 | HIV |
|  | T cells CD29+/CD8+/CD45RA- | HIV vs. control | 180 | <0.0001 | 567 | 170 | HIV |
|  | T cells CD29+/CD8+/CD45RA- | HIV vs. control | 365 | <0.0001 | 535.6 | 170 | HIV |
|  | T cells CD29+/CD8+/CD45RO+ | HIV vs. control | 56 | <0.0001 | 1155 | 204 | HIV |
|  | T cells CD29+/CD8+/CD45RO+ | HIV vs. control | 180 | <0.0001 | 722.4 | 204 | HIV |
|  | T cells CD29+/CD8+/CD45RO+ | HIV vs. control | 365 | <0.0001 | 725.4 | 204 | HIV |
|  | T cells CD29+/CD8+/CD45RO- | HIV vs. control | 56 | 0.0001 | 640.2 | 344 | HIV |
|  | T cells CD8+/CD29+ | HIV vs. control | 56 | <0.0001 | 1546 | 388 | HIV |
|  | T cells CD8+/CD29+ | HIV vs. control | 180 | <0.0001 | 1125 | 388 | HIV |
|  | T cells CD8+/CD29+ | HIV vs. control | 365 | <0.0001 | 1014 | 388 | HIV |
|  | T cells CD8+/CD29- | HIV vs. control | 56 | 0.0016 | 96.6 | 175 | control |
|  | T cells CD8+/CD29- | HIV vs. control | 180 | 0.0001 | 66 | 175 | control |
|  | T cells CD8+/CD29- | HIV vs. control | 365 | <0.0001 | 43.2 | 175 | control |
|  | T cells CD8+/CD127+ | HIV vs. control | 56 | <0.0001 | 175.2 | 323 | control |
|  | T cells CD8+/CD127+ | HIV vs. control | 365 | 0.0002 | 187.2 | 323 | control |
| Additional cytotoxic T cell subsets | T cells CD8+/CD25- | HIV vs. control | 56 | <0.0001 | 1738 | 479 | HIV |
|  | T cells CD8+/CD25- | HIV vs. control | 180 | <0.0001 | 1141 | 479 | HIV |
|  | T cells CD8+/CD25- | HIV vs. control | 365 | <0.0001 | 1010 | 479 | HIV |
|  | T cells CD8+/CD127- | HIV vs. control | 56 | <0.0001 | 1562 | 180 | HIV |
|  | T cells CD8+/CD127- | HIV vs. control | 180 | <0.0001 | 889.5 | 180 | HIV |
|  | T cells CD8+/CD127- | HIV vs. control | 365 | <0.0001 | 777.6 | 180 | HIV |
| NK-T cells | T cells CD16-/CD56+/CD3+/CD117- | HIV vs. control | 180 | 0.0023 | 23.8 | 48 | control |
|  | T cells CD16+/CD56+/CD3+/CD117- | HIV vs. control | 56 | <0.0001 | 0 | 2 | control |
|  | T cells CD16+/CD56+/CD3+/CD117- | HIV vs. control | 180 | 0.0006 | 0 | 2 | control |
|  | T cells CD16+/CD56+/CD3+/CD117- | HIV vs. control | 365 | 0.0002 | 0 | 2 | control |
| Total NK cells | NK cells CD3-/CD56+/CD16+ | HIV vs. control | 180 | <0.0001 | 100.8 | 216 | Control |
| NK cells expressing markers of inhibition | NK cells CD3-/CD56+/CD16+/CD158b+ | HIV vs. control | 180 | 0.0002 | 19.5 | 48 | control |
|  | NK cells CD3-/CD56+/CD16+/CD159a+ | HIV vs. control | 180 | <0.0001 | 35 | 65 | control |
|  | NK cells CD3-/CD56+/CD16+/CD159a+ | HIV vs. control | 365 | <0.0001 | 28 | 65 | control |
| NK cells expressing markers of activation or degranulation | NK cells CD3-/CD56+/CD16-/CD63+ | HIV vs. control | 56 | <0.0001 | 8.4 | 16 | control |
|  | NK cells CD3-/CD56+/CD16-/CD63+ | HIV vs. control | 180 | 0.0015 | 11.7 | 16 | control |
|  | NK cells CD3-/CD56+/CD16-/CD63+ | HIV vs. control | 365 | 0.0001 | 8.8 | 16 | control |
|  | NK cells CD3-/CD56+/CD16-/CD314+ | HIV vs. control | 365 | 0.001 | 8.4 | 2 | HIV |
|  | NK cells CD3-/CD56+/CD16+/CD314+ | HIV vs. control | 56 | <0.0001 | 32 | 137 | control |
|  | NK cells CD3-/CD56+/CD16+/CD314+ | HIV vs. control | 180 | <0.0001 | 26.4 | 137 | control |
|  | NK cells CD3-/CD56+/CD16+/CD314+ | HIV vs. control | 365 | <0.0001 | 48 | 137 | control |
|  | NK cells CD3-/CD56+/CD16+/CD63+/CD314+ | HIV vs. control | 56 | <0.0001 | 3.6 | 16 | control |
|  | NK cells CD3-/CD56+/CD16+/CD63+/CD314+ | HIV vs. control | 180 | 0.0001 | 4.5 | 16 | control |
|  | NK cells CD3-/CD56+/CD16+/CD63+/CD314+ | HIV vs. control | 365 | <0.0001 | 2.7 | 16 | control |
|  | NK cells CD3-CD56+/CD16-/CD69+ | HIV vs. control | 56 | 0.0022 | 1.5 | 2 | control |
| Additional NK cell subsets | NK cells CD16+/CD56+/CD3-/CD117- | HIV vs. control | 56 | 0.0005 | 103.6 | 151 | control |
|  | NK cells CD16+/CD56+/CD3-/CD117- | HIV vs. control | 180 | <0.0001 | 55.2 | 151 | control |
|  | NK cells CD16+/CD56+/CD3-/CD117- | HIV vs. control | 365 | 0.0001 | 61.2 | 151 | control |
| Additional lymphocytes | Lymphocytes CD16-/CD56-/CD3-/CD117- | HIV vs. control | 56 | <0.0001 | 68 | 315 | control |
|  | Lymphocytes CD16-/CD56-/CD3-/CD117- | HIV vs. control | 180 | <0.0001 | 138.6 | 315 | control |

**Supplemental Table S1.** Wilcoxon rank-sum test comparison between the HIV(+) and HC cohorts across 100 immune marker combinations. Comparisons were made at each of the time points (56, 180, and 365 days) for the HIV(+) patients. For the 100 marker panel, false discovery rate was controlled for, and significant differences are indicated by p<0.0033 (1/300 comparisons=0.0033).

| **Funtion** | **biomarker combination** | **compare** | **day** | **p value** | **Median** | | | **higher count** |
| --- | --- | --- | --- | --- | --- | --- | --- | --- |
|  |  |  |  |  | **HIV(-)** | **HIV(+)** | **HC** |  |
| Activated B cells | B cells CD19+/CD80+ | HIV-NEG vs. HIV-POS | 56 | <0.0001 | 2.8 | 0 | . | HIV(-) |
|  | B cells CD19+/CD80+ | HIV-POS vs. control | 56 | <0.0001 | . | 0 | 2 | control |
|  | B cells CD19+/CD80+ | HIV-NEG vs. HIV-POS | 180 | <0.0001 | 4.2 | 0 | . | HIV(-) |
|  | B cells CD19+/CD80+ | HIV-POS vs. control | 180 | 0.0001 | . | 0 | 2 | control |
|  | B cells CD19+/CD80+ | HIV-NEG vs. HIV-POS | 365 | 0.0002 | 2.8 | 0 | . | HIV(-) |
|  | B cells CD19+/CD80+ | HIV-POS vs. control | 365 | 0.0002 | . | 0 | 2 | control |
|  | B cells CD19+/CD86+ | HIV-NEG vs. HIV-POS | 56 | <0.0001 | 5.4 | 0 | . | HIV(-) |
|  | B cells CD19+/CD86+ | HIV-POS vs. control | 56 | <0.0001 | . | 0 | 3 | control |
|  | B cells CD19+/CD86+ | HIV-NEG vs. HIV-POS | 180 | 0.0003 | 4.65 | 1.7 | . | HIV(-) |
|  | B cells CD19+/CD86+ | HIV-POS vs. control | 180 | 0.001 | . | 1.7 | 3 | control |
| T cells expressing markers of activation or degranulation | T cells CD3+/CD69+ | HIV-NEG vs. control | 56 | 0.0058 | 42 | . | 24 | HIV(-) |
|  | T cells CD3+/CD69+ | HIV-NEG vs. HIV-POS | 56 | 0.0028 | 42 | 85 | . | HIV(+) |
|  | T cells CD3+/CD69+ | HIV-POS vs. control | 56 | <0.0001 | . | 85 | 24 | HIV(+) |
|  | T cells CD3+/CD134+ | HIV-NEG vs. HIV-POS | 56 | <0.0001 | 40.7 | 7 | . | HIV(-) |
|  | T cells CD3+/CD134+ | HIV-POS vs. control | 56 | <0.0001 | . | 7 | 20 | control |
|  | T cells CD3+/CD134+ | HIV-NEG vs. HIV-POS | 180 | 0.0004 | 29.35 | 6.8 | . | HIV(-) |
|  | T cells CD3+/CD134+ | HIV-POS vs. control | 180 | 0.0006 | . | 6.8 | 20 | control |
|  | T cells CD3+/CD134+ | HIV-NEG vs. HIV-POS | 365 | 0.0003 | 23.6 | 5.2 | . | HIV(-) |
|  | T cells CD3+/CD134+ | HIV-POS vs. control | 365 | <0.0001 | . | 5.2 | 20 | control |
|  | T cells CD3+/HLA-DR+ | HIV-NEG vs. control | 56 | <0.0001 | 196 | . | 34 | HIV(-) |
|  | T cells CD3+/HLA-DR+ | HIV-NEG vs. HIV-POS | 56 | <0.0001 | 196 | 751 | . | HIV(+) |
|  | T cells CD3+/HLA-DR+ | HIV-POS vs. control | 56 | <0.0001 | . | 751 | 34 | HIV(+) |
|  | T cells CD3+/HLA-DR+ | HIV-NEG vs. HIV-POS | 180 | 0.0006 | 67.5 | 216 | . | HIV(+) |
|  | T cells CD3+/HLA-DR+ | HIV-POS vs. control | 180 | <0.0001 | . | 216 | 34 | HIV(+) |
|  | T cells CD3+/HLA-DR+ | HIV-POS vs. control | 365 | <0.0001 | . | 203 | 34 | HIV(+) |
|  | T cells CD3+/CD134+/CD69-/HLA-DR+ | HIV-NEG vs. control | 56 | <0.0001 | 3.9 | . | 0 | HIV(-) |
|  | T cells CD3+/CD134+/CD69-/HLA-DR+ | HIV-NEG vs. HIV-POS | 56 | 0.0006 | 3.9 | 1.6 | . | HIV(-) |
|  | T cells CD3+/CD134+/CD69-/HLA-DR+ | HIV-NEG vs. control | 180 | <0.0001 | 1.9 | . | 0 | HIV(-) |
|  | T cells CD3+/CD134+/CD69-/HLA-DR+ | HIV-NEG vs. HIV-POS | 180 | 0.0004 | 1.9 | 0 | . | HIV(-) |
|  | T cells CD3+/CD134+/CD69-/HLA-DR+ | HIV-NEG vs. HIV-POS | 365 | 0.0013 | 1.2 | 0 | . | HIV(-) |
|  | T cells CD3+/CD69+/CD134+/HLA-DR+ | HIV-NEG vs. control | 56 | 0.0001 | 0 | . | 0 | HIV(-) |
|  | T cells CD3+/CD69+/CD134+/HLA-DR+ | HIV-NEG vs. HIV-POS | 56 | 0.0022 | 0 | 0 | . | HIV(-) |
|  | T cells CD3+/CD107a/b+ | HIV-NEG vs. control | 56 | 0.0007 | 4.8 | . | 0 | HIV(-) |
|  | T cells CD3+/CD80+ | HIV-NEG vs. HIV-POS | 56 | 0.0003 | 4.2 | 0.7 | . | HIV(-) |
|  | T cells CD3+/CD80+ | HIV-NEG vs. HIV-POS | 180 | 0.0009 | 4 | 1.2 | . | HIV(-) |
|  | T cells CD3+/CD86+ | HIV-NEG vs. control | 56 | <0.0001 | 20.8 | . | 4 | HIV(-) |
|  | T cells CD3+/CD86+ | HIV-POS vs. control | 56 | <0.0001 | . | 31.5 | 4 | HIV(+) |
|  | T cells CD3+/CD86+ | HIV-NEG vs. HIV-POS | 180 | 0.0024 | 6.15 | 9.4 | . | HIV(+) |
|  | T cells CD3+/CD86+ | HIV-POS vs. control | 180 | <0.0001 | . | 9.4 | 4 | HIV(+) |
|  | T cells CD3+/CD86+ | HIV-POS vs. control | 365 | 0.0003 | . | 7.5 | 4 | HIV(+) |
|  | T cells CD3+/CD314+ | HIV-POS vs. control | 365 | 0.0002 | . | 846 | 426 | HIV(+) |
| T cells expressing marker of inhibition | T cells CD3+/ CD159a+ | HIV-NEG vs. control | 180 | 0.0006 | 18.8 | . | 40 | control |
| Cytotoxic T cells | T cells CD8+/CD25+ | HIV-NEG vs. control | 56 | <0.0001 | 6 | . | 0 | HIV(-) |
|  | T cells CD8+/CD25+ | HIV-NEG vs. HIV-POS | 56 | <0.0001 | 6 | 0 | . | HIV(-) |
|  | T cells CD8+/CD25+ | HIV-NEG vs. control | 180 | <0.0001 | 4.15 | . | 0 | HIV(-) |
|  | T cells CD8+/CD25+ | HIV-NEG vs. HIV-POS | 180 | <0.0001 | 4.15 | 0 | . | HIV(-) |
|  | T cells CD8+/CD25+ | HIV-NEG vs. control | 365 | 0.0012 | 4.1 | . | 0 | HIV(-) |
|  | T cells CD8+/CD25+ | HIV-NEG vs. HIV-POS | 365 | <0.0001 | 4.1 | 0 | . | HIV(-) |
|  | T cells CD8+/CD25- | HIV-NEG vs. HIV-POS | 56 | <0.0001 | 581.1 | 1738 | . | HIV(+) |
|  | T cells CD8+/CD25- | HIV-POS vs. control | 56 | <0.0001 | . | 1738 | 479 | HIV(+) |
|  | T cells CD8+/CD25- | HIV-NEG vs. HIV-POS | 180 | <0.0001 | 434 | 1141 | . | HIV(+) |
|  | T cells CD8+/CD25- | HIV-POS vs. control | 180 | <0.0001 | . | 1141 | 479 | HIV(+) |
|  | T cells CD8+/CD25- | HIV-POS vs. control | 365 | <0.0001 | . | 1010 | 479 | HIV(+) |
| Total cssytotoxic NK cells | NK cells CD3-/CD56+/CD16+ | HIV-POS vs. control | 180 | <0.0001 | . | 101 | 216 | control |
| NK cells expressing markers of activation or degranulation | NK cells CD3-/CD16+/CD56+/CD107a/b+ | HIV-NEG vs. control | 56 | <0.0001 | 3.2 | . | 0 | HIV(-) |
|  | NK cells CD3-/CD16+/CD56+/CD107a/b+ | HIV-NEG vs. HIV-POS | 56 | <0.0001 | 3.2 | 0 | . | HIV(-) |
|  | NK cells CD3-/CD16+/CD56+/CD107a/b+ | HIV-NEG vs. control | 180 | <0.0001 | 1.6 | . | 0 | HIV(-) |
|  | NK cells CD3-/CD16+/CD56+/CD107a/b+ | HIV-NEG vs. HIV-POS | 180 | 0.0001 | 1.6 | 0 | . | HIV(-) |
|  | NK cells CD3-/CD16+/CD56+/CD107a/b+ | HIV-NEG vs. HIV-POS | 365 | 0.0002 | 0.7 | 0 | . | HIV(-) |
|  | NK cells CD3-/CD56+16+/ CD314+ | HIV-NEG vs. control | 56 | 0.0017 | 237.6 | . | 137 | HIV(-) |
|  | NK cells CD3-/CD56+16+/ CD314+ | HIV-NEG vs. HIV-POS | 56 | <0.0001 | 237.6 | 32 | . | HIV(-) |
|  | NK cells CD3-/CD56+16+/ CD314+ | HIV-POS vs. control | 56 | <0.0001 | . | 32 | 137 | control |
|  | NK cells CD3-/CD56+16+/ CD314+ | HIV-NEG vs. HIV-POS | 180 | <0.0001 | 165.5 | 26.4 | . | HIV(-) |
|  | NK cells CD3-/CD56+16+/ CD314+ | HIV-POS vs. control | 180 | <0.0001 | . | 26.4 | 137 | control |
|  | NK cells CD3-/CD56+16+/ CD314+ | HIV-NEG vs. HIV-POS | 365 | 0.0015 | 168 | 48 | . | HIV(-) |
|  | NK cells CD3-/CD56+16+/ CD314+ | HIV-POS vs. control | 365 | <0.0001 | . | 48 | 137 | control |
| NK cells expressing markers of inhibition | NK cells CD16+/CD56+/CD159a+/CD3- | HIV-NEG vs. control | 56 | 0.0001 | 148.5 | . | 65 | HIV(-) |
|  | NK cells CD16+/CD56+/CD159a+/CD3- | HIV-NEG vs. HIV-POS | 56 | <0.0001 | 148.5 | 58.8 | . | HIV(-) |
|  | NK cells CD16+/CD56+/CD159a+/CD3- | HIV-NEG vs. HIV-POS | 180 | 0.0003 | 81 | 35 | . | HIV(-) |
|  | NK cells CD16+/CD56+/CD159a+/CD3- | HIV-POS vs. control | 180 | <0.0001 | . | 35 | 65 | control |
|  | NK cells CD16+/CD56+/CD159a+/CD3- | HIV-NEG vs. HIV-POS | 365 | 0.0029 | 72.35 | 28 | . | HIV(-) |
|  | NK cells CD16+/CD56+/CD159a+/CD3- | HIV-POS vs. control | 365 | <0.0001 | . | 28 | 65 | control |

**Supplemental Table S2.** Wilcoxon rank-sum test comparison between the HIV(+), HIV(-) and HC cohorts across 18 immune marker combinations. Comparisons were made at each of the time points (56, 180, and 365 days) for the HIV patients. For the 18 marker panel, false discovery rate was controlled for, and significant differences are indicated by p<0.006 (1/162 comparisons=0.006).

| **Function** | **biomarker combination** | **compare** | **day** | **p value** | **Median** | | **higher count** |
| --- | --- | --- | --- | --- | --- | --- | --- |
|  |  |  |  |  | **HIV(+)** | **HIV(-)** |  |
| T cells expressing markers of activation | T cells CD3+/CD69+ | HIV-NEG vs. HIV-POS | 56 | 0.0054 | 42 | 132 | HIV(+) |
|  | T cells CD3+/CD134+ | HIV-NEG vs. HIV-POS | 56 | 0.0028 | 40.7 | 5.4 | HIV(-) |
|  | T cells CD3+/CD134+ | HIV-NEG vs. HIV-POS | 180 | 0.0165 | 29.35 | 7.15 | HIV(-) |
|  | T cells CD3+/CD134+ | HIV-NEG vs. HIV-POS | 365 | 0.0022 | 23.6 | 4.9 | HIV(-) |
|  | T cells CD3+/HLA-DR+ | HIV-NEG vs. HIV-POS | 56 | 0.0002 | 196 | 1074 | HIV(+) |
|  | T cells CD3+/HLA-DR+ | HIV-NEG vs. HIV-POS | 180 | 0.0017 | 67.5 | 303 | HIV(+) |
|  | T cells CD3+/CD134+/CD69-/HLA-DR+ | HIV-NEG vs. HIV-POS | 365 | 0.016 | 1.2 | 0 | HIV(-) |
|  | T cells CD3+/CD80+ | HIV-NEG vs. HIV-POS | 180 | 0.0097 | 4 | 1.2 | HIV(-) |
| Cytotoxic T cells | T cells CD8+/CD25+ | HIV-NEG vs. HIV-POS | 56 | 0.0016 | 6 | 0 | HIV(-) |
|  | T cells CD8+/CD25+ | HIV-NEG vs. HIV-POS | 180 | <0.0001 | 4.15 | 0 | HIV(-) |
|  | T cells CD8+/CD25+ | HIV-NEG vs. HIV-POS | 365 | 0.0013 | 4.1 | 0 | HIV(-) |
|  | T cells CD8+/CD25- | HIV-NEG vs. HIV-POS | 56 | 0.0001 | 581.1 | 2012 | HIV(+) |
|  | T cells CD8+/CD25- | HIV-NEG vs. HIV-POS | 180 | 0.0007 | 434 | 887 | HIV(+) |
| B cells expressing markers of activation | B cells CD19+/CD80+ | HIV-NEG vs. HIV-POS | 180 | 0.0009 | 4.2 | 1.2 | HIV(-) |
|  | B cells CD19+/CD80+ | HIV-NEG vs. HIV-POS | 365 | 0.0019 | 2.8 | 0 | HIV(-) |
|  | B cells CD19+/CD86+ | HIV-NEG vs. HIV-POS | 56 | 0.0094 | 5.4 | 1.6 | HIV(-) |
|  | B cells CD19+/CD86+ | HIV-NEG vs. HIV-POS | 365 | 0.0159 | 5.15 | 1.15 | HIV(-) |
| NK cells expressing markers of inhibition | NK cells CD16+/CD56+/CD159a+/CD3- | HIV-NEG vs. HIV-POS | 56 | 0.0001 | 148.5 | 56 | HIV(-) |
|  | NK cells CD16+/CD56+/CD159a+/CD3- | HIV-NEG vs. HIV-POS | 180 | 0.0002 | 81 | 24.7 | HIV(-) |
|  | NK cells CD16+/CD56+/CD159a+/CD3- | HIV-NEG vs. HIV-POS | 365 | 0.0111 | 72.35 | 26.3 | HIV(-) |
| NK cells expressing markers of activation or degranulation | NK cells CD3-/CD16+/CD56+/CD107a/b+ | HIV-NEG vs. HIV-POS | 56 | 0.0001 | 3.2 | 0 | HIV(-) |
|  | NK cells CD3-/CD16+/CD56+/CD107a/b+ | HIV-NEG vs. HIV-POS | 180 | 0.0028 | 1.6 | 0 | HIV(-) |
|  | NK cells CD3-/CD16+/CD56+/CD107a/b+ | HIV-NEG vs. HIV-POS | 365 | 0.008 | 0.7 | 0 | HIV(-) |
|  | NK cells CD3-/CD56+16+/ CD314+ | HIV-NEG vs. HIV-POS | 56 | <0.0001 | 237.6 | 35.2 | HIV(-) |
|  | NK cells CD3-/CD56+16+/ CD314+ | HIV-NEG vs. HIV-POS | 180 | <0.0001 | 165.5 | 22.1 | HIV(-) |
|  | NK cells CD3-/CD56+16+/ CD314+ | HIV-NEG vs. HIV-POS | 365 | 0.0046 | 168 | 49.5 | HIV(-) |

**Supplemental Table S3.** Wilcoxon rank-sum test comparison between the HIV(+) patients with Hodgkin's lymphoma, HIV(-) patients and healthy control cohorts across 18 immune marker combinations. Comparisons were made at each of the time points (56, 180, and 365 days). Controlling the false discovery rate gives a significance cutoff at p<0.0185 (1/54 comparisons).

| **Antibody** | | **Source** | **Clone** |
| --- | --- | --- | --- |
| CD45 | ECD | Beckman Coulter | J33 |
| CD14 | FITC | Beckman Coulter | MY4 |
| CD13 | PE | Beckman Coulter | L138 |
| HLA DR | PC5 | Beckman Coulter | IMMU-357 |
| CD3 | FITC | BD | SK7 |
| CD56/16 | PE | BD | MY31/B73.1 |
| CD19 | PC5 | Beckman Coulter | J4.119 |
| HLA DR | FITC | Beckman Coulter | B812.2 |
| CD69 | PE | Beckman Coulter | TP1.55.3 |
| CD3 | ECD | Beckman Coulter | UCHT1 |
| CD134 | PC5 | BD | ACT35 |
| CD45 | PC7 | Beckman Coulter | J33 |
| CD45RA | FITC | Beckman Coulter (Cyto-Stat) | 2H4LDH11LDB9 |
| CD27 | PE | BD | M-T271 |
| CD45RO | ECD | Beckman Coulter | UCHL1 |
| CD4 | PC5 | Beckman Coulter | 13B8.2 |
| CD8 | PC7 | Beckman Coulter | SFCI21Thy2D3 |
| CD29 | PE | Beckman Coulter | 4B4LDC9LDH8 |
| CD193 | FITC | R&D Systems | 61828 |
| CD294 | PE | Miltenyi | BM16 |
| CD4 | ECD | Beckman Coulter | SFCI12T4D11 |
| CD183 | PC5 | BD | 1C6/CXR3 |
| CD45 | PC5 | Beckman Coulter | IMMU19.2 |
| CD194 | PC7 | BD | 1G1 |
| CD80 | FITC | Beckman Coulter | MAB104 |
| CD86 | PE | Beckman Coulter | HA5.2B7 |
| CD19 | ECD | Beckman Coulter | HD237 |
| CD3 | PC5 | Beckman Coulter | UCHT1 |
| TCR γ/δ | FITC | Miltenyi | 11F2 |
| TCR α/β | PE | Miltenyi | BW242/412 |
| CD8 | FITC | Beckman Coulter | SFCI21Thy2D3 |
| CD127 | PE | Beckman Coulter | R34.34 |
| CD25 | PC5 | Beckman Coulter | B1.49.9 |
| CD3 | FITC | Beckman Coulter | UCHT1 |
| CD158b | PE | Beckman Coulter | GL183 |
| CD69 | ECD | Beckman Coulter | TP1.55.3 |
| CD56 | PC5 | Beckman Coulter | N901 (NKH1) |
| CD16 | PC5 | Beckman Coulter | 3G8 |
| CD107a | FITC | BD | H4A3 |
| CD107b | FITC | BD | H4B4 |
| CD159a | PE | Beckman Coulter | Z199 |
| CD63 | FITC | BD | H5C6 |
| CD314 | PE | Beckman Coulter | ON72 |
| CD16 | FITC | Beckman Coulter | 3G8 |
| CD56 | PE | Beckman Coulter | N901 |
| CD117 | PC5 | Beckman Coulter | 104D2D1 |
| CD31 | PE | BD | WM59 |
| CD49a | PE | BD | SR84 |

**Supplemental Table S4.** Antibody vendors and clones used for flow cytometry.
