# Supplementary figures and images for: Immune Recovery Following Autologous Hematopoietic Stem Cell Transplantation in HIV-Related Lymphoma Patients on the BMT CTN 0803/AMC 071 Trial
Source: Front Immunol. 2021 Sep 3;12:700045. doi: 10.3389/fimmu.2021.700045 (PMC8446430; doi:10.3389/fimmu.2021.700045)

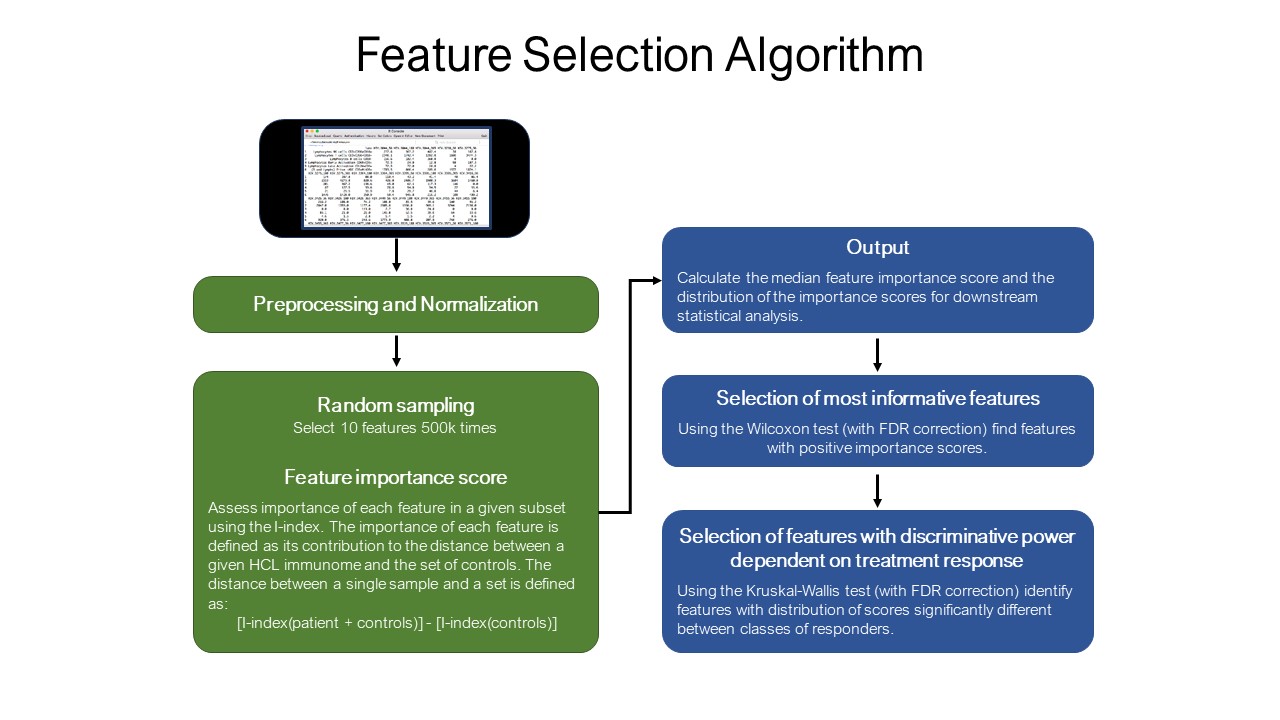

Supplement: Supplementary file 2 [file Image_1.jpeg]

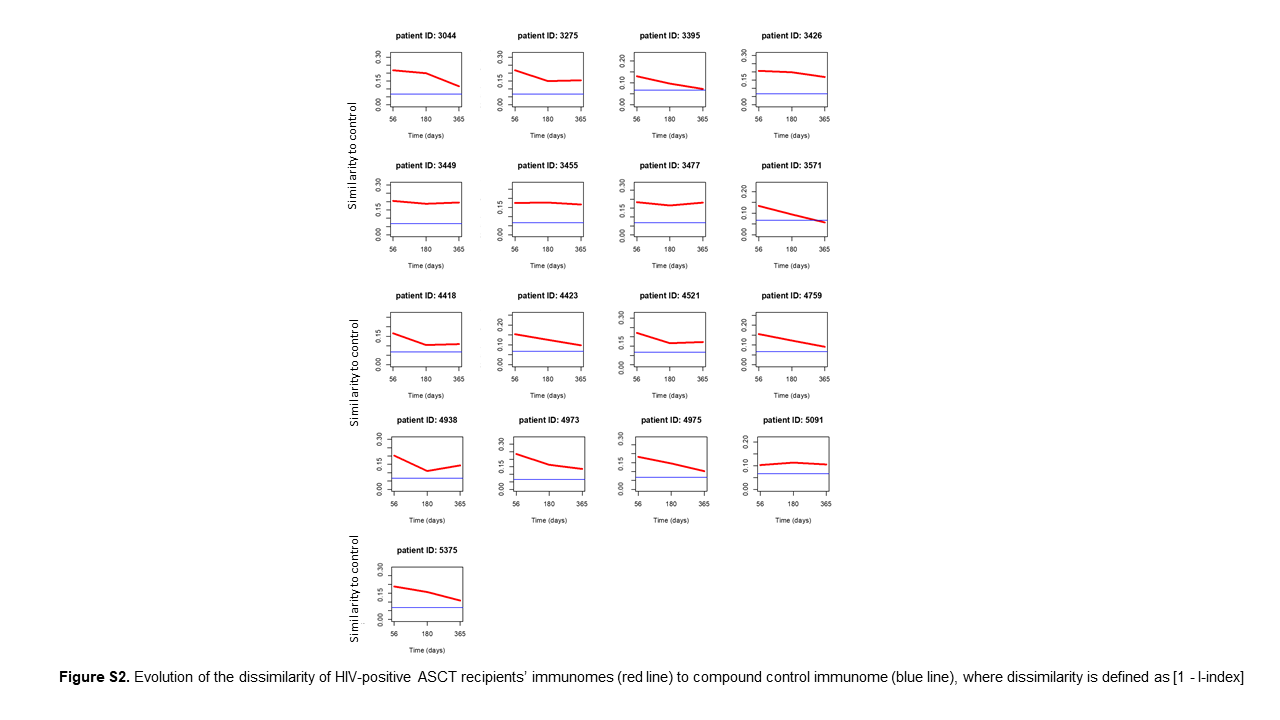

Supplement: Supplementary file 3 [file Image_2.tif]

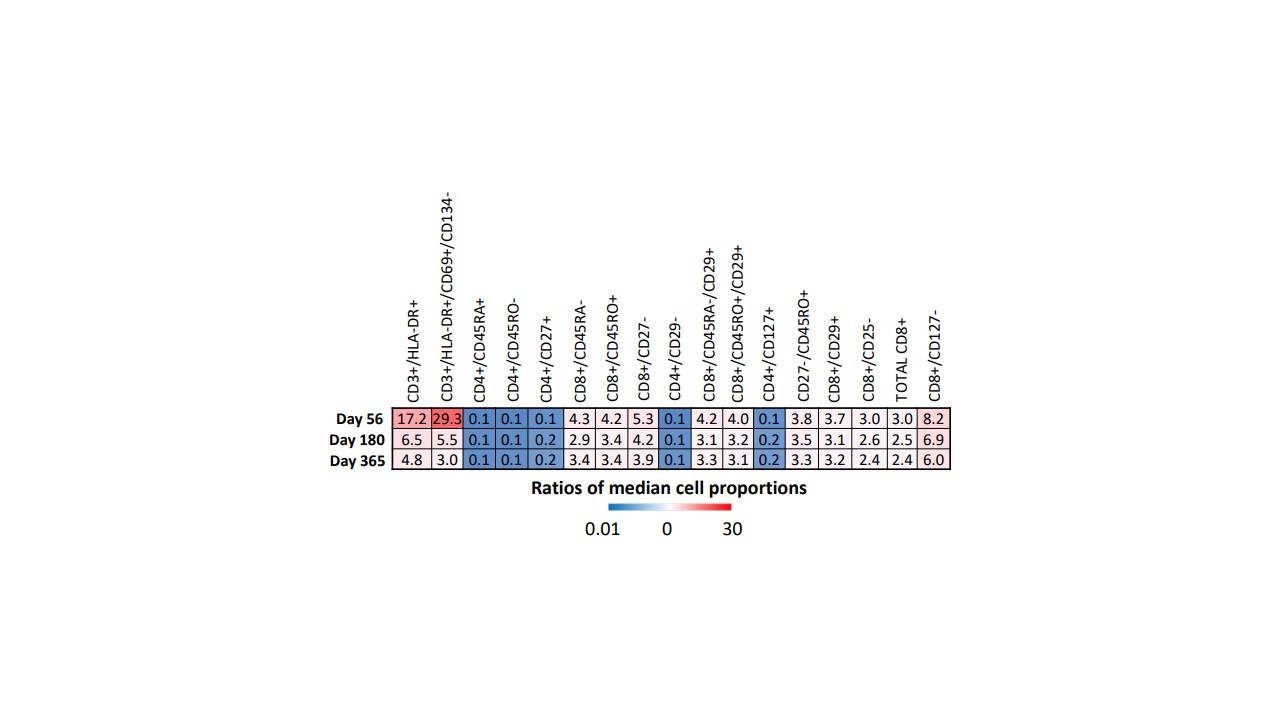

Supplement: Supplementary file 4 [file Image_3.jpeg]
